# Cofilin hyperphosphorylation triggers TDP-43 pathology in sporadic amyotrophic lateral sclerosis

**DOI:** 10.1093/brain/awag096

**Published:** 2026-03-10

**Authors:** Cyril Jones Jagaraj, Sayanthooran Saravanabavan, Sonam Parakh, Mrithulabashini Jayakumar, Sara Assar Kashani, Sina Shadfar, Prachi Mehta, Shashi Gautam, Fabiha Farzana, Alexandra K Suchowerska, Kuok Yap, Marta Vidal, Audrey M G Ragagnin, Md Shafi Jamali, Shu Yang, Thomas Fath, David Craik, Julie D Atkin

**Affiliations:** MND Research Centre, Macquarie Medical School, Faculty of Medicine, Health and Human Sciences, Macquarie University, Sydney, NSW 2109, Australia; MND Research Centre, Macquarie Medical School, Faculty of Medicine, Health and Human Sciences, Macquarie University, Sydney, NSW 2109, Australia; MND Research Centre, Macquarie Medical School, Faculty of Medicine, Health and Human Sciences, Macquarie University, Sydney, NSW 2109, Australia; MND Research Centre, Macquarie Medical School, Faculty of Medicine, Health and Human Sciences, Macquarie University, Sydney, NSW 2109, Australia; MND Research Centre, Macquarie Medical School, Faculty of Medicine, Health and Human Sciences, Macquarie University, Sydney, NSW 2109, Australia; MND Research Centre, Macquarie Medical School, Faculty of Medicine, Health and Human Sciences, Macquarie University, Sydney, NSW 2109, Australia; MND Research Centre, Macquarie Medical School, Faculty of Medicine, Health and Human Sciences, Macquarie University, Sydney, NSW 2109, Australia; MND Research Centre, Macquarie Medical School, Faculty of Medicine, Health and Human Sciences, Macquarie University, Sydney, NSW 2109, Australia; MND Research Centre, Macquarie Medical School, Faculty of Medicine, Health and Human Sciences, Macquarie University, Sydney, NSW 2109, Australia; Translational Neuroscience Facility and Department of Physiology, School of Biomedical Sciences, University of New South Wales, Sydney, NSW 2052, Australia; Institute of Molecular Bioscience, Australian Research Council Centre of Excellence for Innovations in Peptide and Protein Science, The University of Queensland, Brisbane, QLD 4072, Australia; MND Research Centre, Macquarie Medical School, Faculty of Medicine, Health and Human Sciences, Macquarie University, Sydney, NSW 2109, Australia; MND Research Centre, Macquarie Medical School, Faculty of Medicine, Health and Human Sciences, Macquarie University, Sydney, NSW 2109, Australia; MND Research Centre, Macquarie Medical School, Faculty of Medicine, Health and Human Sciences, Macquarie University, Sydney, NSW 2109, Australia; MND Research Centre, Macquarie Medical School, Faculty of Medicine, Health and Human Sciences, Macquarie University, Sydney, NSW 2109, Australia; Dementia Research Centre, Macquarie Medical School, Faculty of Medicine, Health and Human Sciences, Macquarie University, Sydney, NSW 2109, Australia; Institute of Molecular Bioscience, Australian Research Council Centre of Excellence for Innovations in Peptide and Protein Science, The University of Queensland, Brisbane, QLD 4072, Australia; MND Research Centre, Macquarie Medical School, Faculty of Medicine, Health and Human Sciences, Macquarie University, Sydney, NSW 2109, Australia

**Keywords:** TDP-43 pathology, LIMK1 dysregulation, Cofilin hyperphosphorylation and dysregulation, ALS/MND, Actin dysregulation

## Abstract

Pathological forms of TAR-binding protein 43 (TDP-43), involving its aberrant mislocalization to the cytoplasm, inclusion formation, hyperphosphorylation and fragmentation, are present in ∼45%–50% frontotemporal dementia and Alzheimer’s disease individuals and most (97%) amyotrophic lateral sclerosis (ALS) cases. Hence, identifying mechanisms that induce TDP-43 pathology are central to neurodegeneration and developing new therapeutic targets in these conditions. Cofilin is a multifunctional protein with a crucial role in regulating the actin cytoskeleton. Actin has important neuronal-specific activities in dendritic spines, axonal growth cones and synapses and it is in constant equilibrium between two forms: monomeric globular actin (G-actin) and polymeric filamentous actin (F-actin). Cofilin controls actin dynamics by depolymerizing and severing actin filaments. When cofilin is phosphorylated (at serine-3, Ser3) by LIM kinase1 (LIMK1), it becomes inactive, leading to production of more F-actin. Defects in cofilin are well described in other neurodegenerative disorders, unlike in ALS.

We examined phosphorylation of cofilin and actin dynamics in post-mortem spinal cord tissue from sporadic ALS (SALS) patients, the TDP-43 rNLS8 transgenic mouse model and NSC-34 motor neuronal cells expressing cytoplasmic TDP-43. F-actin was pharmacologically stabilized to mimic cofilin hyperphosphorylation and TDP-43 pathology was assessed. Neuronal cells were treated with a non-phosphorylatable cofilin S3A peptide (MAAGVAVSDGVIKVFN) and TDP-43 pathology and apoptosis were evaluated.

Here, we show that cofilin is hyperphosphorylated in human ALS and disease models compared to controls. This was detected in spinal motor neurons from SALS patients and a TDP-43 mouse model (rNLS8) displaying key ALS phenotypes, and in motor neuronal NSC-34 cells expressing cytoplasmic TDP-43. Supporting this observation, more F-actin relative to G-actin was present in cortical/spinal cord lysates from SALS patients and TDP-43 rNLS8 mice, and NSC-34 cells expressing TDP-43. We also show that mimicking cofilin hyperphosphorylation by pharmacological stabilization of F-actin induced TDP-43 pathology: cytoplasmic mislocalization, inclusion formation, hyperphosphorylation and fragmentation, and promoted its recruitment into stress granules (SGs). Furthermore, we detected increased levels of LIMK1 phosphorylation and tropomyosin isoforms 4.1 and 4.2 in SALS patients. These findings reveal aberrant cofilin hyperphosphorylation disrupts actin dynamics, triggering TDP-43 pathology and SG recruitment in SALS. They imply that preventing cofilin phosphorylation is a novel therapeutic strategy applicable to most ALS cases. Treatment of neuronal cells with the S3A peptide prevented features of TDP-43 pathology and apoptosis compared to control peptides. These findings thus describe a novel pathogenic mechanism producing TDP-43 pathology, applicable to most ALS cases and other neurodegenerative diseases.

## Teaser

Jones Jagaraj *et al.* show that cofilin is hyperphosphorylated in post-mortem spinal cord tissue from patients with sporadic ALS, disrupting actin dynamics and promoting TAR-binding protein 43 (TDP-43) mislocalization and aggregation. Preventing cofilin phosphorylation *in vitro* reduced TDP-43 pathology, highlighting a potential new therapeutic strategy.

## Introduction

Amyotrophic lateral sclerosis (ALS) is a fatal, rapidly progressing neurodegenerative disorder affecting motor neurons (MNs) that overlaps clinically and pathologically with frontotemporal dementia (FTD).^1^ Almost all ALS (97%) and ∼50% FTD and Alzheimer’s disease cases display pathological forms of TAR DNA-binding protein 43 (TDP-43).^2,3^ TDP-43 is an RNA/DNA binding protein normally localized to the nucleus, but in pathological conditions, it undergoes phosphorylation, fragmentation, cytoplasmic mislocalization and aggregation.^4,5^ TDP-43 pathology is strongly linked to toxicity and neurodegeneration,^6^ thus it is central to ALS/FTD. TDP-43 is also recruited to stress granules (SGs) in ALS: heterogeneous transient structures composed of proteins, pre-mRNAs and untranslated mRNA transcripts, associated with neurodegeneration.^7^ Therefore, understanding the mechanisms that induce TDP-43 pathology is critically important in neurodegeneration.

Cofilin is a multifunctional protein essential for regulating the actin cytoskeleton, and in neurons it supports dendritic spine organization, axonal branching, and synaptic signalling.^8-10^ Actin is highly abundant and its functions include vesicle transport and muscle contraction.^11^ Actin exists in either monomeric, globular form (G-actin) or polymerized into short (5 nm diameter), linear filaments (F-actin) that generate movement and transport cargo.^12^ Cofilin regulates actin dynamics—the steady-state assembly/disassembly of F-actin filaments^13^—by depolymerizing and severing these filaments, leading to less polymerized F-actin.^13^ Cofilin is tightly regulated by phosphorylation (serine-3, Ser3) by LIMK domain kinase I (LIMK1)^14^ , which inactivates cofilin and hence induces actin polymerization,^15^ whereas dephosphorylation activates its function, leading to less F-actin.^14^ The activity of LIMK1 is in turn regulated by its phosphorylation at Thr 508,^16,17^ which inactivates cofilin. Tropomyosins also regulate actin^18,19^ and tropomyosin 4.1 and 4.2 (Tpm-4.1/4.2) are actin-stabilizing proteins that promote cofilin phosphorylation and thus actin polymerization.^20^

Cofilin abnormalities, leading to dysfunctional actin dynamics, are well documented in other neurodegenerative disorders such as Alzheimer’s,^21^ Parkinson’s^22^ and Huntington’s diseases^23^ and spinal muscular atrophy^24^ but are neglected in ALS. Cofilin is linked to many processes associated with neurodegeneration^25^ including redox sensing,^26^ apoptosis,^27^ DNA repair,^28^ cellular transport,^29^ autophagy,^27^ lipid metabolism,^30^ maintenance of nuclear architecture^31^ and aging.^32^ Interestingly, genetic variants in *PFN-1* encoding profilin are present in familial ALS patients, which catalyses actin polymerization and thus has opposing functions to cofilin.^33^ However, the role of cofilin in ALS in the context of TDP-43 pathology is unexplored.

Here, we demonstrate that cofilin is aberrantly hyperphosphorylated in ventral horn MNs and cortical/spinal cord lysates from sporadic ALS patients and a TDP-43 mouse model (NEFH-tTA/tetO-hTDP-43ΔNLS; ‘rNLS’),^34^ displaying ALS phenotypes including TDP-43 pathology.^34^ We also detected cofilin hyperphosphorylation in MN-like NSC-34 cells expressing cytoplasmic TDP-43 compared to controls. This was accompanied by dysregulation of actin dynamics, increased phosphorylation of LIMK1 and increased Tpm-4.1/4.2. Pharmacological induction of actin polymerization, mimicking cofilin hyperphosphorylation, induced features of TDP-43 pathology and its recruitment to SGs. Treatment of neuronal cells with a cofilin peptide that cannot be phosphorylated prevented features of TDP-43 pathology, directly linking cofilin phosphorylation with TDP-43 pathology. These findings reveal new insights into sporadic ALS (SALS) pathophysiology, identifying aberrant cofilin hyperphosphorylation as trigger of TDP-43 pathology and a novel therapeutic target.

## Materials and methods

### Constructs and cell lines

Constructs and cell lines used in this study are detailed in Supplementary Table 1.

### Ethics statement

All animal husbandry and experimental procedures were performed in compliance with the Animal Ethics Committee, Macquarie University (ARA #2015/042, ARA #2017/019) and the Internal Biosafety Committee, Macquarie University (NLRD 5201401007, 5974 52019597412350). All human studies were approved by Macquarie University Human Ethics (HEC #5201600387 and #5201700825).

### Animals

TDP-43 rNLS8 mice were fed with chow containing 200 mg/kg doxycycline (Dox) prior to experimentation to suppress human TDP-43 transgene expression (*hTDP-43^ΔNLS^*).^34^ During experimentation, mice were switched to normal chow (lacking Dox) to induce *hTDP-43^ΔNLS^* expression. Mice (∼8–10 weeks age) were housed in temperature and humidity-controlled conditions (21 ± 1°C, 55 ± 5%) with a 12 h light/dark cycle. They were randomly allocated to groups for all experiments. Both sexes were included and balanced between groups. Littermate non-transgenic and monogenic animals were used as controls.

### TDP-43 rNLS8 mouse cortex lysate preparation

Mice were anaesthetized with a mixture of isoflurane and oxygen and transcardially perfused with ∼20 ml of 0.9% sodium chloride solution. The brain was removed, bisected through the midline and immediately stored at −80°C. The cortices were lysed in radioimmunoprecipitation assay (RIPA) buffer (50 mm Tris–HCl, pH 7.5, 150 mm NaCl, 0.1% (w/v) SDS, 1% (w/v) sodium deoxycholate and 1% (v/v) Triton X-100 with 1% (v/v) protease and phosphatase inhibitor cocktail). Samples were sonicated, spun at 4°C and ∼100 000*g* for 30 min and supernatant stored at −80°C. Total protein concentration was quantified (Pierce™ BCA Protein Assay Kit), following the manufacturer’s instructions.

### Human spinal cord lysate preparation

Human lumbar spinal cord segments (L3–L5) from ALS patients and control individuals without neurological/psychiatric disease were provided by the Victorian Brain Bank (Supplementary Table 2). Tissues (100 mg) were lysed in RIPA buffer. After homogenization, samples were centrifuged for 10 min at 100 000*g*, and the supernatant frozen at −80°C. Protein lysates were quantified using the bicinchoninic acid (BCA) assay following the manufacturer’s instructions. The patients were classified as sporadic because no previous family history of ALS or related neurodegenerative disorders was reported by the patients or referring clinicians. However, as the patients were not screened genetically, we cannot rule out the possibility that unknown mutations were present. There are no reported gender differences in actin dynamics between males and females.^35,36^

### Cell lysate preparation

Cells were lysed with RIPA buffer and incubated on ice for 20 min. Following centrifugation at 12 000 rpm for 10 min at 4°C the RIPA-soluble fraction (supernatant) was collected as the lysate.

### Soluble and insoluble fraction preparation

SH-SY5Y cells were treated with 1% dimethyl sulfoxide (DMSO), or 1 µM jasplakinolide for 1 h and lysed with RIPA buffer and incubated on ice for 20 min. Following centrifugation at 100 000*g* for 30 min at 4°C the RIPA-soluble fraction (supernatant) was collected as the soluble fraction. The pellet was washed twice with RIPA buffer to remove residual soluble proteins and urea buffer [7 M urea, 2 M thiourea, 4% 3-[(3-cholamidopropyl)dimethylammonio]-1-propanesulfonate (CHAPS) and 30 mM Tris] was added at a volume of 5× weight/volume of pellet followed by sonication and centrifugation at 100 000*g* at 4°C for 30 min. The supernatants were retained as the insoluble protein fractions.

### G-actin/F-actin *in vivo* assays

#### Cell lysates

F- and G-actin fractions from lysates were prepared using a G-Actin/F-Actin *in vivo* assay Biochem kit (Cytoskeleton) following manufacturer’s instructions. Briefly, transfected cells were washed in PBS and incubated in 200 μl of lysis buffer (LAS2) at 37°C for 10 min. Lysates (100 μl) were centrifuged at 500*g* for 5 min at 37°C to remove debris, and supernatant was centrifuged ∼100 000*g* for 1 h at 37°C. The G-actin rich supernatant and F-actin rich pellet were collected: 100 μl F-actin depolymerizing buffer was added to the latter followed by incubation on ice for 1 h. The G-actin:F-actin ratio was quantified by western blotting.

#### Mouse cortex tissues

TDP-43 rNLS8 mice and littermate controls were anaesthetized with a mixture of isoflurane and oxygen and intracardially perfused with ∼20 ml 0.9% saline solution. Cortices were dissected, frozen on dry ice and stored at −80°C until required when 50 mg was lysed in 500 µl LAS2 buffer. F/G-actin rich fractions were prepared as above.

#### Human spinal cords (control and SALS patients)

LAS2 buffer (1 ml) was added to 100 mg each spinal cord tissue sample (Supplementary Table 3), and F/G-actin fractions were prepared as above.

### Nuclear and cytoplasmic fractionation

NSC-34 cells were treated with 1% DMSO or 1 µM jasplakinolide in DMSO for 1 h, followed by a PBS wash and resuspension in 500 μl fractionation buffer [10 mM HEPES (pH 7.5), 10 mM KCl, 0.075% 2 mM MgCl_2_, 1 mM EDTA, 1 mM DTT and 0.1% protease and phosphatase inhibitor cocktail]. Cell lysates were passed through a 27G needle 10× until all cells were lysed and then incubated on ice for 20 min. Subsequently, the lysate was centrifuged at 720*g* for 5 min, yielding supernatant containing cytoplasm and the nuclear pellet. The latter was resuspended in 500 μl fractionation buffer, passed through a 27G needle and centrifuged at 720*g* for 10 min. The resulting supernatant was discarded, and the pellet was resuspended in RIPA buffer, followed by sonication (10 cycles), yielding the nuclear fraction. The supernatant obtained from the first centrifugation was centrifuged at 10 000*g* for 10 min. The resulting supernatant was collected as the cytoplasmic fraction.

### Solid-phase peptide synthesis

Cofilin peptides were synthesized by Fmoc solid-phase peptide synthesis using an automated peptide synthesizer on 2-chlorotrityl chloride resin with sidechain-protected amino acid blocks. To generate linear peptides, sidechain protected peptides were cleaved off resin using a TFA (trifluoroacetic acid)/TIPS (triisopropylsilane)/H_2_O (96:2:2 v/v/v) solution for 3 h. The resin-liberated and sidechain-deprotected peptides were precipitated by ice-cold diethyl ether, then pelleted by centrifugation, dissolved in 50% ACN (acetonitrile), 0.1% TFA solution and lyophilized. Peptides were purified using preparative-scale reverse-phase high-performance liquid chromatography using a linear ACN gradient of 10%–50% with fractions collected during chromatography. Fractions were analysed by electrospray ionization mass spectrometry for mass confirmation. The purity of the peptides was verified on an analytical system based on a fixed UV wavelength detector at 215 and 280 nm. Supplementary Table 4 describes the list of peptides synthesized.

### Cofilin peptide treatment

Neuro-2A cells were cultured in Dulbecco’s modified Eagle medium (DMEM) supplemented with 10% fetal bovine serum, 1% penicillin–streptomycin at 37°C in a humidified atmosphere with 5% CO_2_. Cells were seeded in 6-well plates at 2 × 10^5^ cells/well and adhered overnight. Following treatment, they were transfected (1 µg DNA per well) using Lipofectamine 3000 (Thermo Fisher Scientific) according to the manufacturer’s protocol. Cells were incubated with transfection mixture for 6 h, and fresh medium containing 50 µg/ml cofilin peptide applied for 48 h (final concentration 50 µg/ml). Cells were incubated for 24 h for G-actin and F-actin assays as above.

### Antibodies

All primary and secondary antibodies used in immunohistochemistry, immunocytochemistry and western blotting are listed in Supplementary Table 5.

### Immunohistochemistry

Human thoracic spinal cord segments (T4–T5) from three SALS patients (clinical diagnosis confirmed post-mortem, P7–9) and three controls without neurological/psychiatric disease (C5–7) were used (Supplementary Table 2). Sections were pre-heated at 70°C for 30 min and paraffin was removed by washing; 2× xylene, 2× 100% ethanol, 1× 95% ethanol, 1× 70% ethanol and 2× distilled water. Antigen retrieval was performed by boiling sections in 10 mM sodium citrate buffer for 30 min and blocked with 5% goat serum containing 0.1% Tween 20 in PBS (PBST) for 1 h. Primary antibodies were applied in blocking buffer (5% goat serum in PBST) at 4°C overnight, followed by secondary antibodies in the dark at room temperature. Slides were washed with PBST and mounted with anti-fade fluorescent mounting media (Dako) and left to cure overnight before imaging.

### Immunohistochemistry of mouse spinal cords

Frozen lumbar spinal cord sections were equilibrated to room temperature and fixed in 4% paraformaldehyde (PFA) for 10 min at room temperature, then 10 min in pre-chilled methanol at −20°C. Following a PBST rinse, slides were permeabilized and blocked [5% bovine serum albumin (BSA), 1% Triton X in PBS], washed with PBST, and primary antibodies were added overnight at room temperature. After washing with PBST, secondary antibodies were added for 1 h at room temperature. After a final incubation with Hoechst for 10 min, slides were washed with PBST, mounted with anti-fade fluorescent mounting media (Dako) and left overnight before imaging.

### Immunocytochemistry

NSC-34 cells cultured on 13 mm coverslips (Menzel-Glasser) in DMEM were transfected with plasmids using Lipofectamine 2000 (Invitrogen) for 72 h following the manufacturer’s instructions. To induce actin polymerization, cells were treated with 1% DMSO, 1 µM jasplakinolide (Sigma) or 1 µM latrunculin A in DMSO (Sigma) for 1 h, washed in PBS and fixed in 4% PFA for 10 min, permeabilized with 0.1% Triton X-100 in PBS for 5 min and blocked with 5% BSA in PBS for 30 min.

#### SG and importin experiments

Primary antibodies in blocking buffer were incubated with cells at 4°C overnight, coverslips washed with PBS and secondary antibodies were added for 1 h. After three washes in PBS, cells were treated with 0.5 µg/ml Hoechst 33258 and coverslips mounted onto slides in anti-fade fluorescent mounting media (Dako).

### SDS-PAGE and western blotting

Samples (20 µg protein) were subjected to 4%–20% SDS-PAGE (Bio-Rad) and transferred onto nitrocellulose membranes following the manufacturer’s instructions (Bio-Rad). Blots were pre-incubated in blocking solution containing 5% (w/v) skim milk in Tris-buffered saline with 0.1% Tween 20 (TBST), followed by incubation overnight at 4°C in primary antibodies diluted in 5% (w/v) BSA (phospho-antibodies) or 5% (w/v) skim milk in TBST. Immunoreactivity was revealed using a Clarity™ ECL Western Blotting Substrate kit (BioRad) and images obtained using ChemiDoc MP with Image Lab™ software (BioRad). The intensity of each protein band relative to GAPDH, total protein or β-actin was quantified using ImageJ (v. 1.54f; National Institutes of Health). Densitometry measurements of western blots were normalized to loading controls relative to the mean of control samples.

### Confocal microscopy

Human tissue sections/cells were imaged using a 63× oil objective lens and Zeiss LSM 880 inverted confocal laser-scanning microscope. Confocal microscopy of mouse tissues was performed using a Stellaris 5 Confocal Laser Microscope (Leica Biosystems) equipped with a supercontinuum white light laser (440–790 nm). Images were acquired under 63× objective magnification and multiple Z-stacks were obtained to cover tissue depth (0.3 µm intervals). The images at different spectral wavelengths were sequentially acquired in separate tracks to prevent bleed-through effects. Photomultiplier sensitivities and offsets were set to a level in multichannel imaging, at which bleed-through effects from one channel to another were absent. All confocal images were obtained under equivalent conditions of laser power, pinhole size, gain and offset settings between groups. All laser exposures were kept constant between groups.

Quantification of 6+ spinal MNs for each sample was performed using ImageJ (1.54f). MNs were identified by their size, location and morphology, as the largest cells in the spinal cord ventral horn, that stained positive for NeuN or SMI-32. The total corrected fluorescence was calculated by subtracting the total area of each, identified by positive NeuN or SMI-32 staining, and the mean fluorescent background intensity from the integrated density.

### Statistics

Data are presented as mean value with standard deviation (SD). Statistical comparisons between group means were performed using GraphPad Prism 8.4.1 software. Unpaired *t*-test with or without Welch’s correction, one-way/two-way ANOVA, with *post hoc* Tukey’s or Sidak’s, or Kruskal–Wallis test for multiple comparisons were performed. Significance threshold was set at *P* = 0.05. The number of independent repeats (*n*), statistical tests used and statistical significances (*P*-values) are specified. All immunofluorescence and immunohistochemistry experiments were performed blinded.

## Results

### G- and F-actin are dysregulated in SALS

As cofilin modulates actin dynamics, we first examined whether actin is dysregulated in lumbar spinal cords from SALS patients (with no family history of ALS) and non-neurological controls (*n* = 6/group; Supplementary Table 3), by quantifying the proportion of G-actin and F-actin in these tissues. The SALS patients (six males) and control group (five males, one female) had the same mean age, 67.3 years. These samples were age-matched because normal aging impacts actin polymerization.^37^ A G-actin and F-actin assay was performed, which uses distinct buffers to isolate/stabilize G-actin and F-actin fractions (Fig. 1A). Standard lysis buffers cannot be used because they solubilize all proteins indiscriminately.

**Figure 1:**
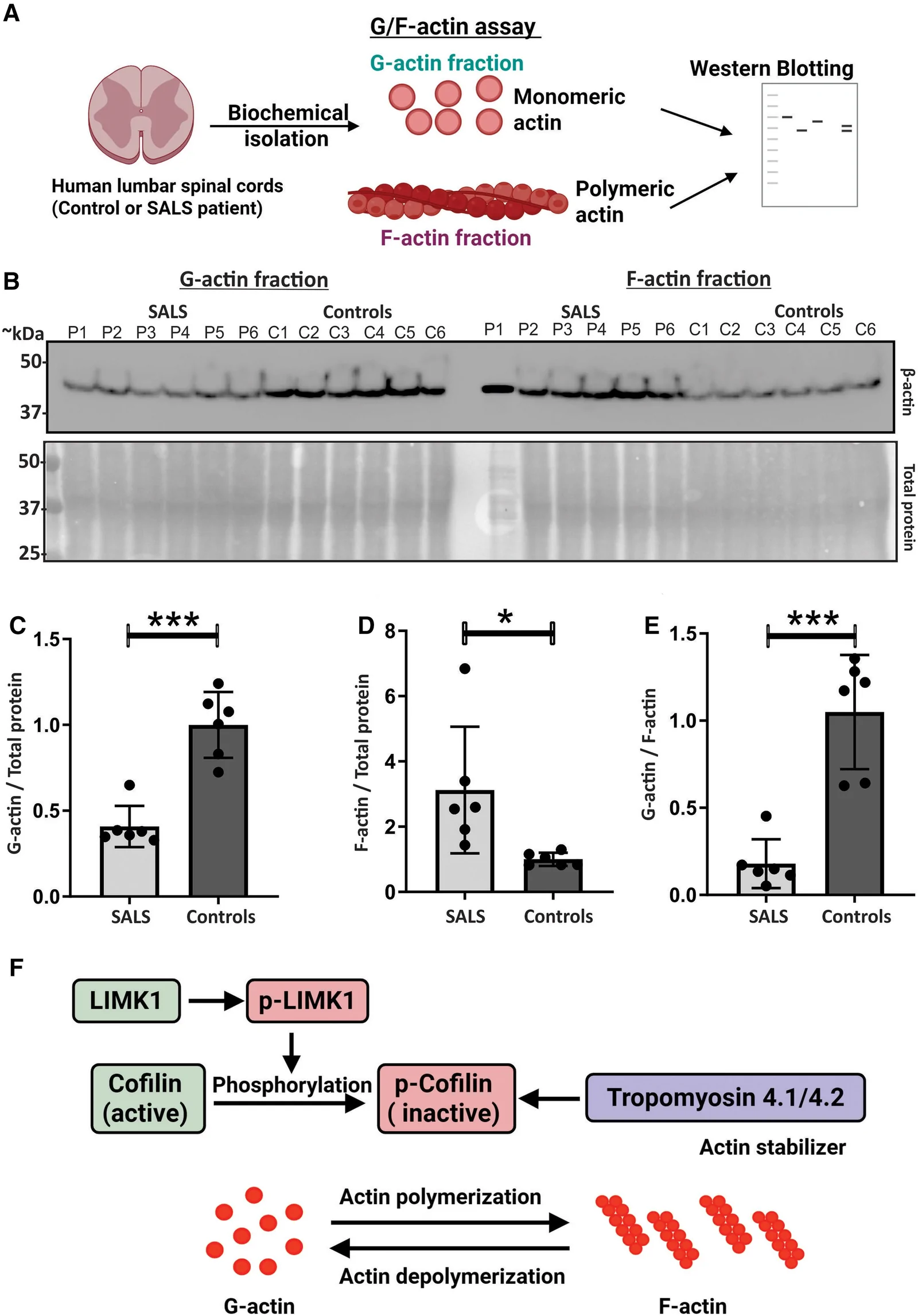
**G and F-actin are dysregulated in spinal cords of SALS patients.** (**A**) Schematic diagram of the G/F-actin *in vivo* assay performed in SALS patients and non-neurological controls. Created in BioRender. Jayakumar, M. (2026) https://BioRender.com/0xofxol. (**B**) Western blotting of G-actin and F-actin fractions of lumbar spinal cord tissues from non-neurological controls and SALS patients (Supplementary Table 3) using an anti-β-actin antibody (*n* = 6 per group). Total protein staining using Ponceau S was used as a loading control. The approximate position of molecular weight markers in kDa are shown on the *left*. (**C**) Quantification of the G-actin fractions of the blots in **B**. Intensity of G-actin normalized to total protein (whole lane quantified) relative to the mean of control in fractions of SALS patients. Data represented as mean ± SD (*n* = 6), unpaired *t*-test with Welch’s correction, two-tailed (2.5-fold, ***P < 0.001). A significant decrease in the levels of G-actin in SALS patients compared to controls was detected. (**D**) Quantification of F-actin fractions of blots shown in **B**. Relative intensity of F-actin normalized to total protein in fractions, mean ± SD (*n* = 6), unpaired *t*-test with Welch’s correction, two-tailed (3.12-fold, *P < 0.05). A significant increase in the levels of F-actin in SALS patients compared to controls was detected. (**E**) Quantification of G-actin/F-actin ratio normalized to total protein from **B**, mean ± SD (*n* = 6), unpaired *t*-test with Welch’s correction, two-tailed (5.58-fold, ***P < 0.001). A significant decrease in the G-actin/F-actin ratio in SALS patients compared to controls was detected. (**F**) Schematic diagram of LIMK1-mediated cofilin-actin dynamics cytoskeletal signalling cascade. SALS = sporadic amyotrophic lateral sclerosis; SD = standard deviation. Created in BioRender. Jayakumar, M. (2026) https://BioRender.com/5swhwcb.

Western blotting for β-actin and quantification of G- and F-actin fractions revealed a significant decrease G-actin in human SALS lysates compared to controls (Fig. 1B and C), demonstrating that less monomeric actin is present. Correspondingly, significantly more F-actin was present in SALS patients (Fig. 1B–D), revealing more polymerized actin. Furthermore, the G:F-actin ratio was significantly decreased in SALS compared to controls (Fig. 1B–E), together implying that actin polymerization is dysregulated in SALS lumbar spinal cords.

### Cofilin phosphorylation is increased in spinal MNs of SALS patients.

We next examined phosphorylated cofilin in SALS patient lumbar spinal cords compared to controls (Supplementary Table 2), using an antibody that detects phosphorylation specifically at Ser3. Lysates were prepared from the same patients and combined with lysates from three additional SALS and three controls (Supplementary Table 2). The resulting nine SALS and controls were analysed in two cohorts based on mean age.^37^ Western blotting of cohort 1 (mean age; control: 75.6 years, SALS: 76.8 years) and cohort 2 (mean age; control: 59.1 years, SALS: 60.3 years) revealed significantly more phosphorylated cofilin in SALS in both cohorts compared to age-matched controls (Fig. 2A–B and E–F). In contrast, there were no differences in total cofilin levels or loading, assessed by GAPDH (Fig. 2A–C and E–G). Hence the phosphorylation state of cofilin, but not overall expression, is altered in SALS, suggesting dysregulation of cofilin activity and actin cytoskeleton dynamics, rather than changes in cofilin abundance *per se*. When results from the two cohorts were combined, similar findings were obtained (Supplementary Fig. 2C and D), suggesting that cofilin dysregulation is a consistent, disease-associated feature in SALS, independent of age.

**Figure 2:**
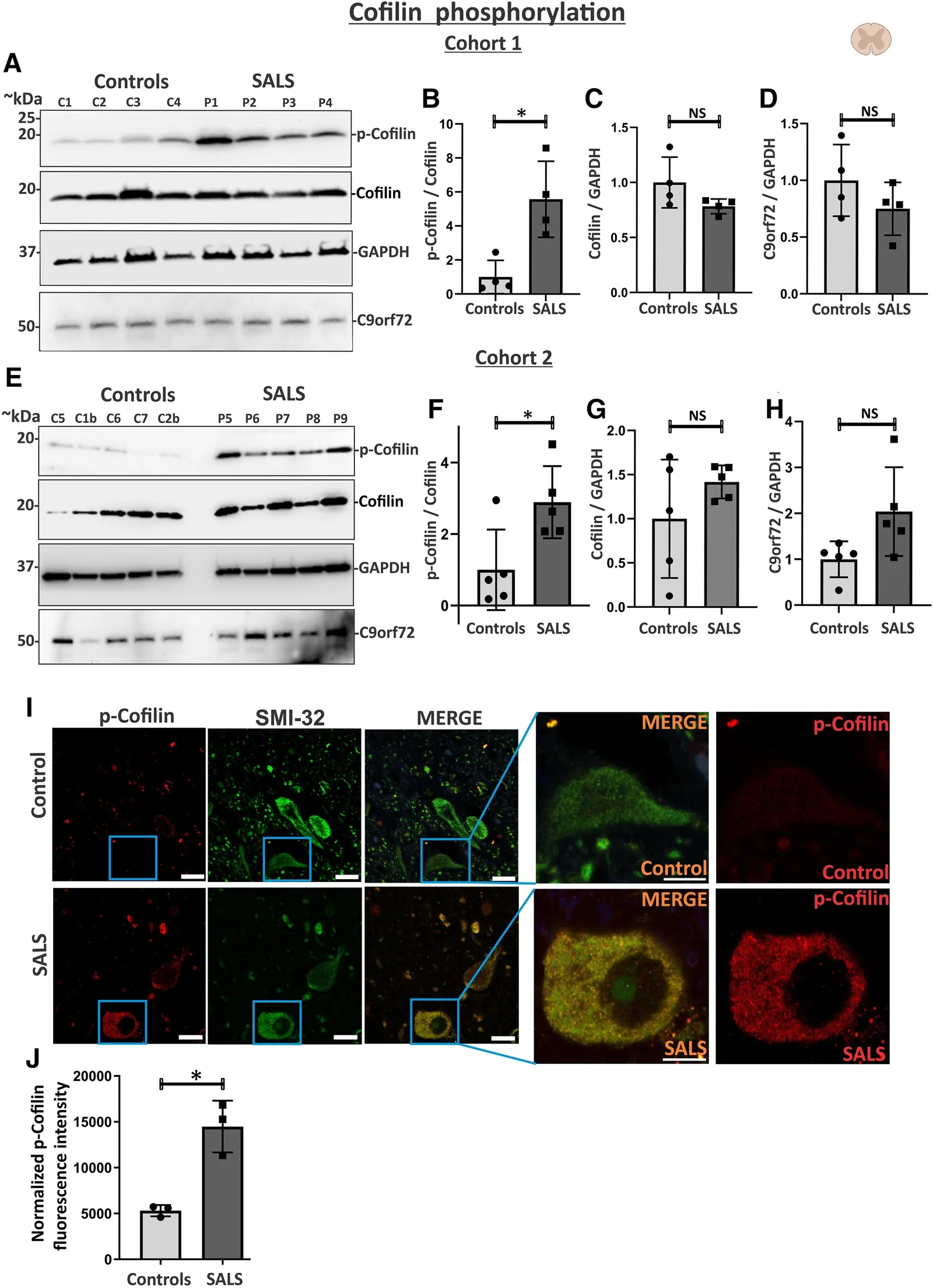
**Cofilin phosphorylation is enhanced in SALS patient spinal cords.** (**A** and **E**) Western blotting using anti-phospho-cofilin (Ser3), anti-cofilin, anti-C9orf72 and anti-GAPDH antibodies of lumbar spinal cord lysates prepared from human SALS patients and controls. (**A**) Cohort 1 (*n* = 4 per group, controls and SALS patients), (**E**) cohort 2 (*n* = 5 per group, controls and SALS patients (Supplementary Table 2). (**B**) Quantification of cohort 1 western blots shown in **A**. Intensity of phosphorylated cofilin normalized to total cofilin in spinal cords of SALS patients, relative to the mean of controls. Data are represented as mean ± SD (*n* = 4, per group, controls and SALS patients), unpaired *t*-test with Welch’s correction, two-tailed (5.5-fold, *P < 0.05). Spinal cord icon was created in BioRender. Jayakumar, M. (2026) https://BioRender.com/om4t94t. (**C** and **D**) Quantification of cohort 1 western blots shown in **A**. Intensity of total cofilin and C9orf72 normalized to GAPDH in spinal cords of SALS patients, relative to the mean of controls. Data are represented as mean ± SD, *n* = 4, unpaired *t*-test with Welch’s correction, two-tailed. (**F**) Quantification of cohort 2 western blots shown in **E**. Intensity of phosphorylated cofilin normalized to total cofilin in spinal cords of SALS patients, relative to the mean of controls. Data are represented as mean ± SD, *n* = 5 (per group, controls and SALS patients), unpaired *t*-test with Welch’s correction, two-tailed (2.89-fold, *P < 0.05). (**G** and **H**) Quantification of cohort 2 western blots shown in **E**. Intensity of total cofilin and C9orf72 normalized to GAPDH in spinal cords of SALS patients, relative to the mean of controls. Data are represented as mean ± SD, *n* = 5, unpaired *t*-test with Welch’s correction, two-tailed. A significant increase in the phosphorylation of cofilin in human SALS patients compared to controls was detected in both cohorts 1 and 2. The same membrane was sequentially reprobed with different primary antibodies, allowing detection of multiple target proteins on the same blot. (**I**) Immunohistochemistry of post-mortem spinal cord sections of three SALS patients and three control patients using antibodies against phospho-cofilin (Ser3) and SMI-32. Phosphorylated-cofilin was expressed diffusely in both control and in SALS patient motor neurons. Scale bar = 20 μm. Magnified view of the region indicated by the blue box shows a representative single neuron. Scale bar = 10 μm. (**J**) Quantification of normalized p-cofilin fluorescent intensities revealed increased phospho-cofilin in SALS patient MNs compared to controls. Data represent at least 6 neurons counted per control or SALS patients, mean ± SD, *n* = 3 (SALS patients or controls), unpaired *t*-test with Welch’s correction, two-tailed, *P < 0.05. MN = motor neuron; NS = non-significant; SALS = sporadic amyotrophic lateral sclerosis; SD = standard deviation.

As cofilin hyperphosphorylation is present in *C9ORF72*-familial ALS^38^ and 5%–10% of SALS patients possess *C9ORF72* mutations, we wanted to exclude the possibility that hyperphosphorylation of cofilin in SALS is due to loss of *C9ORF72*. Western blotting (using a validated antibody)^39^ confirmed there were no differences in C9orf72 levels between SALS lysates(Fig. 2A–D and E–H). The results from both cohorts combined into one group also gave similar findings (Supplementary Fig. 2E). Quantification of phosphorylated cofilin normalized to total C9orf72 revealed a significant increase in SALS spinal cords compared to controls (cohorts 1 and 2; Supplementary Fig. 2F and G). Hence cofilin hyperphosphorylation is robustly elevated in SALS, even after adjusting for C9orf72 levels. Dysregulation of actin dynamics and aberrant cofilin phosphorylation in SALS is therefore not due to *C9ORF72* haploinsufficiency.

Next, we examined whether phosphorylated cofilin was increased specifically in MNs of human SALS spinal cords. Immunohistochemistry and confocal microscopy were performed using SMI-32 (neuronal marker) and phosphorylated cofilin (Ser3-specific) antibodies. MNs were identified by their size, location and morphology—the largest cells in the ventral horn staining positive for SMI-32 (Fig. 2I). The fluorescence intensity of phosphorylated cofilin was quantified and normalized to the cell soma area so that results were not dependent on each MN size. These analyses revealed that levels of phosphorylated cofilin were significantly increased in SALS MNs compared to controls (Fig. 2J).

### LIMK1 phosphorylation and tropomyosin-4.1/4.2 are increased in SALS

We then examined mechanisms leading to cofilin hyperphosphorylation by assessing whether LIMK1 phosphorylation and Tpm-4.1 and Tpm-4.2 are also dysregulated in SALS patient spinal cords (Fig. 1F).

LIMK1 phosphorylation was first investigated in the SALS and control lysates (Supplementary Table 2) by western blotting using a phospho-specific (Thr508) antibody for p-LIMK1. Significantly more phosphorylated LIMK1 was present in SALS compared to controls in both cohorts (Fig. 3A–B and D–E). There was no change in total LIMK1 levels or loading (GAPDH) (Fig. 3A–C and D–F), confirming that phosphorylation of LIMK1 was specifically increased. Hence enhanced phosphorylation of LIMK1 correlates with more cofilin phosphorylation and F-actin in SALS. The results from both cohorts combined into one group were also statistically significant for more phosphorylated LIMK1 (Supplementary Fig. 3C and D).

**Figure 3:**
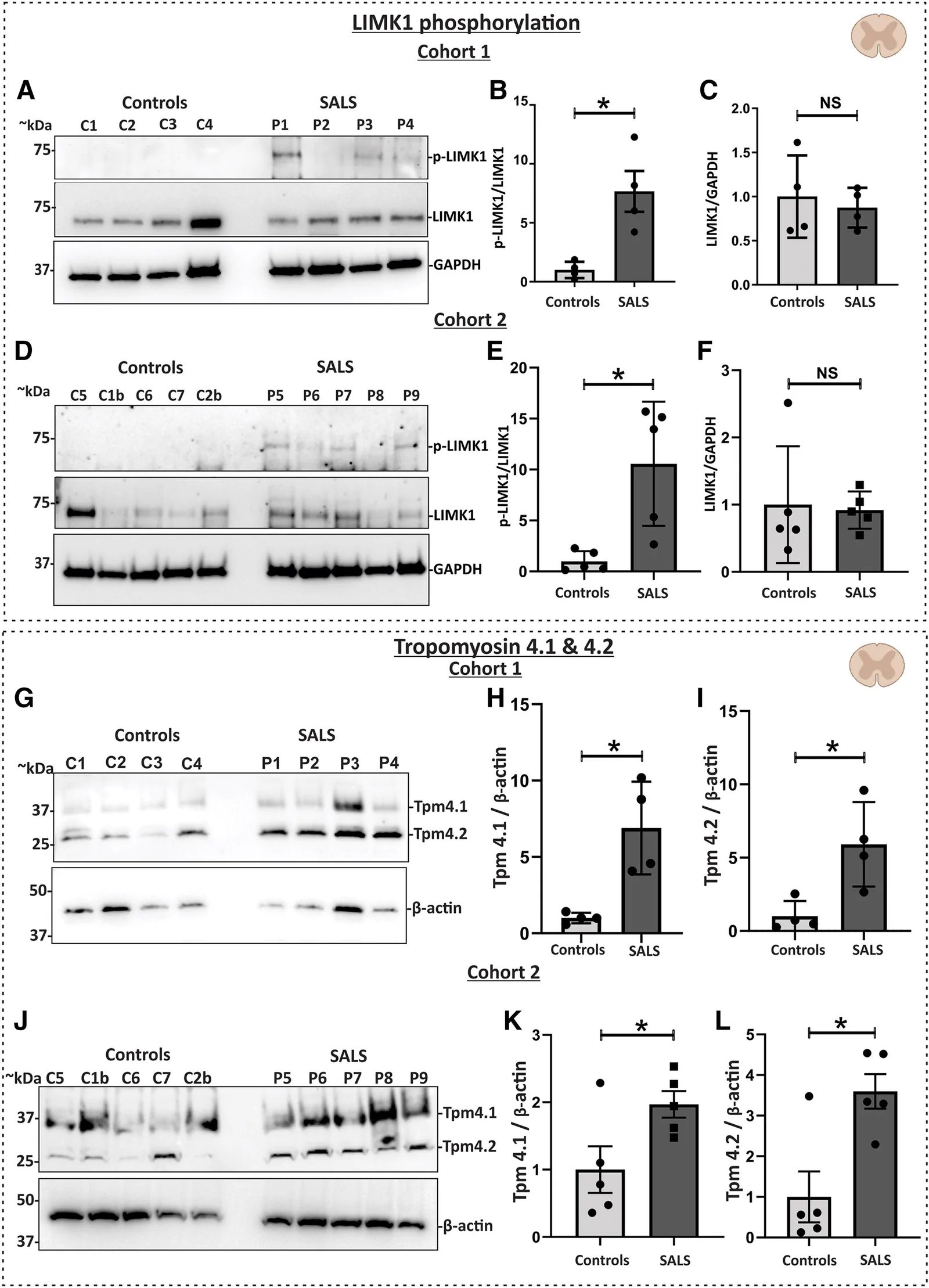
**Phosphorylation of LIMK1 and tropomyosin 4.1 and 4.2 are enhanced in SALS patient spinal cords.** (**A** and **D**) Western blotting using anti-phospho-LIMK1 (Thr508), anti-LIMK1 and anti-GAPDH antibodies in lumbar spinal cord lysates prepared from human SALS patients and controls. (**A**) Cohort 1 (*n* = 4 per group, SALS patients and controls), (**D**) cohort 2 (*n* = 5 per group, SALS patients and controls) (Supplementary Table 2). (**B** and **E**) Quantification of cohort 1 (**B**) and cohort 2 (**E**) western blots shown in **A** and **D**. The graph represents the intensity of phosphorylated LIMK1 normalized to total LIMK1 in spinal cords of SALS patients, relative to the mean of control patients. Data are represented as mean ± SD, unpaired *t*-test with Welch’s correction, two-tailed (cohort 1: 7.66-fold, *P < 0.05; cohort 2: 10.56-fold, *P < 0.05). Significantly more phosphorylated LIMK1 was present in human lumbar SALS patients compared to controls. (**C** and **F**) Quantification of cohort 1 (**C**) and cohort 2 (**F**) western blots shown in **A** and **D**. The graph represents the intensity of LIMK1 normalized to GAPDH in spinal cords of SALS patients, relative to the mean of controls. Data are represented as mean ± SD, unpaired *t*-test with Welch’s correction, two-tailed. The position of approximate molecular weights (in kDa) are shown on the *left* for each blot. GAPDH was used as a loading control. (**G** and **J**) Western blotting of cohort 1 (**G**) and cohort 2 (**J**) using anti-Tpm-4 and anti-β-actin antibodies of lumbar spinal cord lysates prepared from human SALS patients and controls, *n* = 4 and *n* = 5, respectively. The positions of approximate molecular weight markers (in kDa) are shown on the *left*. β-Actin was used as a loading control. The expected molecular weight ranges for these isoforms are approximately 37 kDa for Tpm-4.1 and 25 kDa for Tpm-4.2. (**H** and **I**, and **K** and **L**) Quantification of western blots shown in **G** and **J**. The graph depicts the intensity of Tpm-4.1 and Tpm-4.2 normalized to β-actin in spinal cords of SALS patients relative to the mean of the controls. These analyses revealed significantly more Tpm-4.1 (cohort 1: 6.89-fold, *P < 0.05; cohort 2: 1.96-fold, *P < 0.05) and Tpm-4.2 are present in SALS patient lumbar spinal cords compared to controls (cohort 1: 5.8-fold, *P < 0.05; cohort 2: 3.5-fold, *P < 0.05). Data are represented as mean ± SD, unpaired *t*-test with Welch’s correction, two-tailed. A significant increase in the levels of phosphorylated LIMK1, tropomyosin 4.1 and tropomyosin 4.2 in human SALS patients compared to controls was detected in both cohorts. NS = not significant; SALS = sporadic amyotrophic lateral sclerosis; SD = standard deviation. Spinal cord icon was created in BioRender. Jayakumar, M. (2026) https://BioRender.com/om4t94t.

We next examined Tpm-4.1/4.2 in SALS patients. Western blotting revealed significantly more Tpm-4.1 and Tpm-4.2 in SALS compared to controls (Fig. 3G–I and J–L), consistent with more cofilin phosphorylation and hence polymeric actin. The results from both cohorts combined into one group also gave similar findings (Supplementary Fig. 4C). Together these data show that more phospho-cofilin is associated with dysregulation of LIMK1 and Tpm-4 signalling pathways.

### Cofilin phosphorylation correlates with disease progression and TDP-43 pathology in TDP-43 rNLS8 mice

Next, we examined cofilin phosphorylation in the rNLS8 mouse displaying TDP-43 pathology and key ALS phenotypes: motor neuron loss, impaired motor function and reduced survival.^34^ This involves Dox-suppressible expression of human TDP-43 transgene with nuclear localization signal genetically ablated, driven by the neurofilament heavy chain promoter.^34^ Dox (200 mg/kg) normally added to the diet is removed at ∼8–10 weeks of age to induce TDP-43 expression.^34^ Cytoplasmic TDP-43 accumulates in the cortex and spinal cord of these animals 1 week after Dox removal, when human TDP-43 expression is initiated (Supplementary Fig. 5). At 2 weeks off Dox, TDP-43 pathology and mild motor impairment appears. At 4 weeks, there is major cortical atrophy and muscle NMJ denervation, and by 6 weeks, loss of spinal cord MNs, severe motor phenotype and early death (Supplementary Fig. 5). Surprisingly, these mice undergo partial ‘recovery’ when Dox is returned, manifesting as loss of TDP-43 pathology, prevention of MN loss and rescue of motor impairment.^34^

We next examined cofilin phosphorylation using western blotting of whole cortex tissues from this model and age-matched littermate controls. To determine the required sample size, we performed a power analysis (α = 0.05, power = 80%) based on the human data in Fig. 2, which indicated an observed effect size of ∼1.5. Considering that inbred mice exhibit reduced variablity compared to humans (~50%–75% lower SD), power analysis indicated that a sample size of *n* = 3 mice per group is sufficient, thereby supporting the use of smaller cohorts in the mouse studies.

In the absence of TDP-43 pathology (1 week off Dox) there were no significant differences in cofilin phosphorylation (Fig. 4A and B) or total cofilin (Fig. 4A and Supplementary Fig. 6F) in cortical lysates compared to controls. However, when TDP-43 pathology first appears (2 weeks off Dox), significantly more phosphorylated cofilin (Fig. 4C and D) was present in rNLS8 mice compared to controls. However, there were no differences in total cofilin (Fig. 4C and Supplementary Fig. 6G). Hence, aberrant phosphorylation of cofilin correlates with TDP-43 pathology in the rNLS8 model. Similarly, at both 4 weeks and 6 weeks off Dox, significantly more phosphorylated cofilin was present in rNLS8 mice cortices compared to controls (Fig. 4E–H). Similarly, no changes in total cofilin were detected at these timepoints (Supplementary Fig. 6H and I). Finally, western blotting of lysates from mice at the recovery phase, lacking TDP-43 pathology, displayed no significant differences in rNLS8 mice compared to controls (Fig. 4I and J) and no differences in total cofilin (Fig. 4I and Supplementary Fig. 6J). Hence phosphorylation of cofilin correlates with the appearance of TDP-43 pathology, early in disease course, in an ALS mouse model.^34^

**Figure 4:**
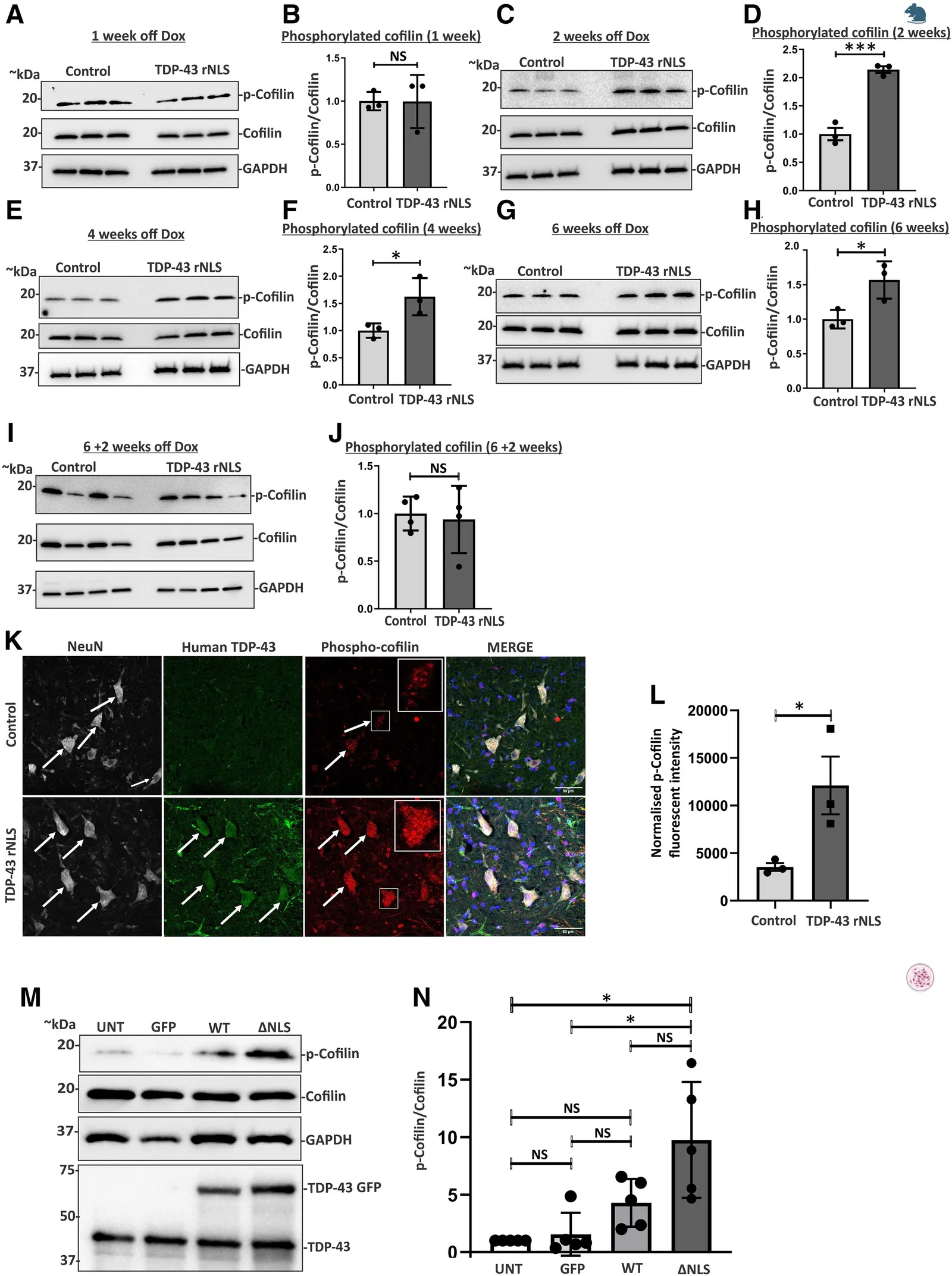
**Cofilin phosphorylation increases with disease progression in TDP-43 rNLS8 mice and in cells expressing cytoplasmic TDP-43**. (**A**, **C**, **E**, **G** and **I**) Western blotting using anti-phospho-cofilin, anti-cofilin and anti-GAPDH antibodies in lysates prepared from the cortex of littermate control and TDP-43 rNLS8 mice (*n* = 3/group) after 1 week off Dox (**A**), 2 weeks off Dox (**C**), 4 weeks off Dox (**E**), 6 weeks off Dox (**G**) and recovery stage (6 weeks off Dox +2 weeks on Dox) (**I**). The approximate molecular weight (kDa), as indicated by molecular weight markers, is shown on the *left*. GAPDH was used as a loading control. (**B**) Quantification of the western blots in **A**. Intensity of phospho-cofilin normalized to total cofilin, relative to the mean of controls. Data are represented as mean ± SD (*n* = 3), unpaired *t*-test, two-tailed. No significant differences were present in 1-week off Dox TDP-43 rNLS8 mice compared to control mice. (**D**) Quantification of western blot in **C**, mean ± SD (*n* = 3) (2.1-fold, ***P < 0.001). (**F**) Quantification of western blot in **E**, mean ± SD (*n* = 3) (1.623-fold, *P < 0.05). (**H**) Quantification of western blot in **G**, mean ± SD (*n* = 3) (1.56-fold, *P < 0.05). (**D**, **F** and **H**) Cofilin phosphorylation is significantly increased in 2, 4 and 6 weeks off Dox TDP-43 rNLS8 mice compared to control mice. (**J**) Quantification of western blots in **I**, mean ± SD (*n* = 3). No significant difference in phosphorylated cofilin in 6 weeks off Dox +2 weeks on Dox TDP-43 rNLS8 mice compared to control mice was detected. (**K**) Immunohistochemistry of spinal cord sections of TDP-43 rNLS8 mice and control mice stained using phospho-cofilin, human TDP-43 and NeuN antibodies. Confocal image (maximum projection of *z*-stacks; 25 × 0.3 µm) shows increased phosphorylated cofilin in cytoplasmic TDP-43 expressing NeuN-positive spinal motor mouse neurons (white arrows). Scale bars = 50 μM. (**L**) Quantification of normalized p-cofilin fluorescent intensities in spinal cords of TDP-43 rNLS8 mice compared to controls. Data are represented as mean ± SD (*n* = 3), unpaired *t*-test, two-tailed; *P < 0.05. (**M**) Western blotting of lysates prepared from untransfected NSC-34 cells (UNT) and cells expressing empty vector, GFP-only (GFP), wildtype TDP-43 or TDP-43ΔNLS, using anti-phospho-cofilin (Ser3), anti-cofilin, anti-GAPDH and anti-TDP-43 antibodies. GAPDH was used as a loading control. (**N**) Quantification of phospho-cofilin normalized to total cofilin, relative to the mean of controls from the western blots in **M**. Data are represented as mean ± SD (*n* = 5), one-way ANOVA, Kruskal–Wallis multiple comparison test; *P < 0.05. A significant increase in phosphorylated cofilin in cells expressing TDP-43-ΔNLS compared to both untransfected cells (9.75-fold, *P < 0.05) and GFP-only controls (6.27-fold, *P < 0.05) was detected. NS = non-significant; SD = standard deviation. Mouse and cell culture plate icon was created in BioRender. Jayakumar, M. (2026) https://BioRender.com/om4t94t.

### Phosphorylated cofilin is increased in spinal MNs of TDP-43 rNLS8 mice

Next, we examined whether phosphorylated cofilin was increased specifically in MNs of TDP-43 rNLS8 mice. Immunohistochemistry of ventral horn sections was performed using anti-phosphorylated cofilin (Ser3), anti-human TDP-43 and anti-NeuN (neuronal marker) antibodies, from TDP-43 rNLS8 mice at 6 weeks off Dox and littermate controls, when significant (30%) loss of spinal MNs is detected.^34^ The human-TDP-43–specific antibody used detects human TDP-43, but not endogenous mouse TDP-43. Motor neurons were identified by their size, location and morphology, as largest NeuN-positive ventral horn cells. The rNLS transgene was expressed predominately in the neuronal cytoplasm, as expected, with little staining in control mice (Fig. 4K). The fluorescence intensity of phosphorylated cofilin in MNs was quantified and normalized to cell soma area (identified by NeuN staining) so that results were not dependent on neuron size. Significantly more phosphorylated-cofilin in spinal MNs of TDP-43 rNLS8 mice compared to control mice (Fig. 4K and L).

### Cytoplasmic TDP-43 increases phosphorylated cofilin

We next examined whether cells expressing pathological TDP-43 also display increased phosphorylated cofilin. Motor neuronal NSC-34 cells expressing green fluorescent protein (GFP)-tagged wildtype TDP-43 (TDP-43^WT^), TDP-43 with nuclear localization signal removed (TDP-43^ΔNLS^, to mislocalize TDP-43 to the cytoplasm and form inclusions, as in SALS), or controls, GFP-only or untransfected cells, were examined 72 h post-transfection.^40^ Microscopy confirmed that TDP-43^ΔNLS^ was expressed as cytoplasmic inclusions (57.67% cells), diffuse in the cytoplasm (34.33%) or in both nucleus and cytoplasm (8% cells). In contrast, TDP-43^WT^ was expressed predominately in the nucleus (89% cells) or in both the nucleus and cytoplasm (11% cells) (Supplementary Fig. 6K and L) and no cytoplasmic inclusions formed.

Western blotting of lysates revealed a significant increase in phosphorylated cofilin in cells expressing TDP-43^ΔNLS^ compared to untransfected and GFP-only cells. In contrast, there was no significant difference between cells expressing TDP-43^WT^ compared to controls, although a trend was evident (Fig. 4M and N). Although not significant, a trend towards more phosphorylated cofilin in TDP-43^ΔNLS^ cells compared to TDP-43^WT^ cells was present. There were no differences between untransfected and GFP-only, demonstrating that cofilin phosphorylation was not dysregulated by non-specific protein overexpression. As a major (ΔNLS) or minor (WT) proportion of TDP-43 was expressed in the cytoplasm, these data imply that cytoplasmic TDP-43 results in hyperphosphorylation of cofilin. We also quantified phosphorylated cofilin normalized to endogenous TDP-43 or overexpressed TDP-43, but similar findings were obtained, demonstrating that differences in transfection efficiency do not impact these results (Supplementary Fig. 6N and O). Hence cytoplasmic mislocalization of TDP-43 leads to elevated cofilin phosphorylation.

### More F-actin is present in TDP-43 rNLS8 mice

Actin was examined next in cortex tissue from rNLS8 mice at 6 weeks off Dox and from littermate controls. A G-actin/F-actin assay and western blotting for β-actin revealed significantly less G-actin levels in rNLS8 mice compared to controls (Fig. 5A and B) demonstrating that less monomeric actin was present. Correspondingly, more polymerized F-actin was present in rNLS8 lysates compared to controls (Fig. 5A and C), and the G/F-actin ratio was significantly reduced (Fig. 5A and D) compared to controls. Expression of the TDP-43 transgene in rNLS8 mice was also confirmed by western blotting (Supplementary Fig. 7B). Therefore, actin dynamics is disturbed in TDP-43 rNLS8 mice cortices.

**Figure 5:**
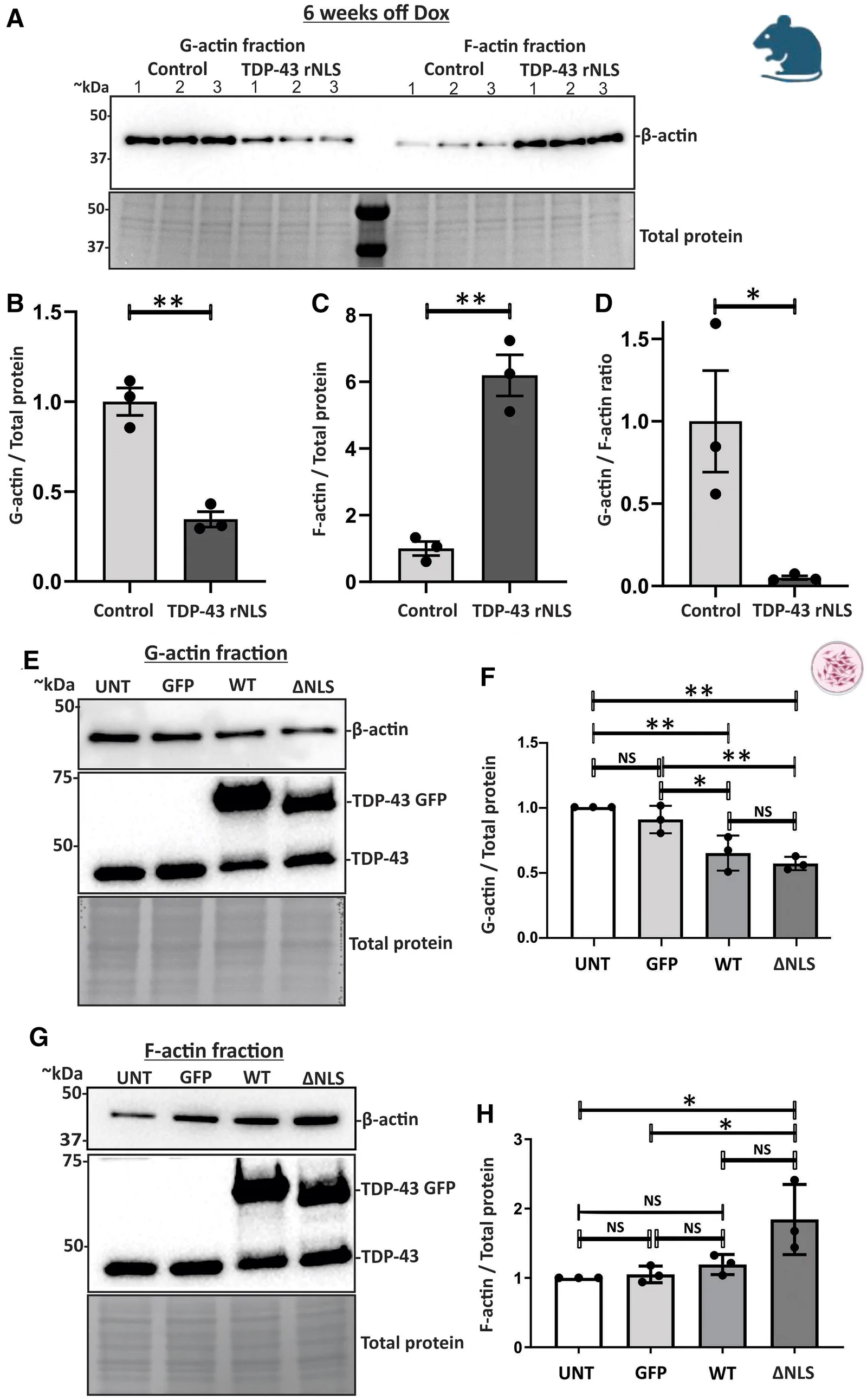
**Actin dynamics is disrupted in TDP-43 rNLS8 mice and cells expressing cytoplasmic TDP-43.** (**A**) Western blotting of G-actin and F-actin fractions prepared from TDP-43 rNLS8 mice and littermate controls at 6 weeks off Dox (late symptomatic stage) using an anti-β-actin antibody. Total protein staining with Ponceau S was used as a loading control. The approximate positions of the molecular weight markers are shown on the *left*. (**B**) Quantifications of G-actin normalized to total protein, relative to the mean of controls from the western blots in **A**, mean ± SD (*n* = 3 mice), unpaired *t*-test, two-tailed (2.89-fold, **P < 0.005). Significantly less G-actin was present in TDP-43 rNLS8 mice relative to control animals. (**C**) Quantification of F-actin normalized to total protein staining from western blots (**A**). Data represented as mean ± SD (*n* = 3), unpaired *t*-test, two-tailed (6.19-fold, **P < 0.005). Significantly more F-actin is present in TDP-43 rNLS8 mice relative to control animals. (**D**) Quantification of G-actin/F-actin ratio from **A**, mean ± SD (*n* = 3), unpaired *t*-test, two-tailed (19.7-fold, *P < 0.05). A significant decrease in the G:F-actin ratio is present in 6 weeks off Dox TDP-43 rNLS8 mice compared to control mice. (**E** and **G**) Western blotting of G-actin and F-actin fractions prepared from untransfected NSC-34 cells (UNT) and cells expressing empty vector, GFP-only (GFP), wildtype TDP-43 or TDP-43ΔNLS, using anti-β-actin and anti-TDP-43 antibodies. Total protein staining with Ponceau S was used as a loading control. (**F** and **H**) Quantification of G-actin and F-actin levels normalized to total protein staining, relative to the mean of untransfected controls from western blots in **E** and **G**. Data are represented as mean ± SD (*n* = 3), one-way ANOVA, Tukey’s multiple comparison test *P < 0.05, **P < 0.005. NS = non-significant; SD = standard deviation; WT = wild-type.Mouse and cell culture plate icons were created in BioRender. Jayakumar, M. (2026) https://BioRender.com/om4t94t.

### Overexpression of cytoplasmic TDP-43 disturbs G:F-actin ratio

Next, a G-actin and F-actin assay was performed on cells, followed by western blotting for β-actin. Quantification revealed significantly less G-actin in cells expressing TDP-43^ΔNLS^ compared to untransfected and GFP-only cells, but not TDP-43^WT^ cells (Fig. 5E and F). Similarly, significantly more F-actin was present in TDP-43^ΔNLS^ compared to untransfected and GFP-only, but not TDP-43^WT^, cells (Fig. 5G and H). Although not statistically significant, trends of more F-actin and less G-actin in TDP-43^ΔNLS^ compared to TDP-43^WT^ cells were evident. There were no differences between GFP only and untransfected cells demonstrating that actin dynamics was not perturbed by non-specific protein overexpression. Significantly less G-actin, but not F-actin, was present in TDP-43^WT^ compared to untransfected and GFP-only cells. Given that either a major (ΔNLS) or minor (WT) proportion of TDP-43 was expressed in the cytoplasm, these data imply that expression of cytoplasmic TDP-43 results in more polymerized actin in ALS. We further quantified the levels of G- and F-actin normalized to endogenous TDP-43 or overexpressed TDP-43 with the same result (Supplementary Fig. 7E and F). Hence these results link cytoplasmic TDP-43 to actin dysregulation in ALS.

### Actin polymerization induces TDP-43 pathology

We next examined whether induction of F-actin pharmacologically can initiate TDP-43 pathology. NSC-34 cells were treated with jasplakinolide (1 µM for 1 h), a widely used, potent inducer of actin nucleation that stimulates actin polymerization.^41^ Control cells were treated for 1 h with either DMSO (vehicle) or 1 µM latrunculin A, which inhibits actin polymerization and has the opposite effect to jasplakinolide. A G-actin and F-actin assay confirmed that jasplakinolide induced actin polymerization and latrunculin A induced actin depolymerization (Supplementary Fig. 8A). No obvious signs of toxicity of jasplakinolide or latrunculin A were evident by light microscopy (normal morphology and few apoptotic nuclei).

We examined both overexpressed TDP-43 and endogenous TDP-43 in cells. NSC-34 cells expressing GFP-tagged TDP-43^WT^ at 72 h post-transfection were treated with either 1 µM jasplakinolide, 1 µM latrunculin A or DMSO (vehicle) for 1 h, then examined by confocal fluorescence microscopy for GFP expression. Cells were classified as mislocalized to the cytoplasm when TDP-43 was present in cytoplasm only, or cytoplasm and nucleus. Cells were classified as nuclear when TDP-43 was present only in the nucleus. In DMSO vehicle and latrunculin-treated cells, <13% of cells displayed cytoplasmic, mislocalized TDP-43 (Fig. 6A and B). However, significantly more cells expressed cytoplasmic TDP-43 following jasplakinolide treatment compared to both DMSO and latrunculin A (Fig. 6A and B). Quantification of TDP-43 inclusions, detected by the presence of green-fluorescent, compact, cytoplasmic TDP-43 aggregates visible by microscopy, revealed significantly more TDP-43 inclusions following jasplakinolide treatment compared to DMSO or latrunculin A treatment (Fig. 6A and C). Hence jasplakinolide, which produces more F-actin, induces TDP-43 mislocalization and inclusion formation. Latrunculin A had no effect, thus perturbation of actin dynamics *per se* does not induce these defects.

**Figure 6:**
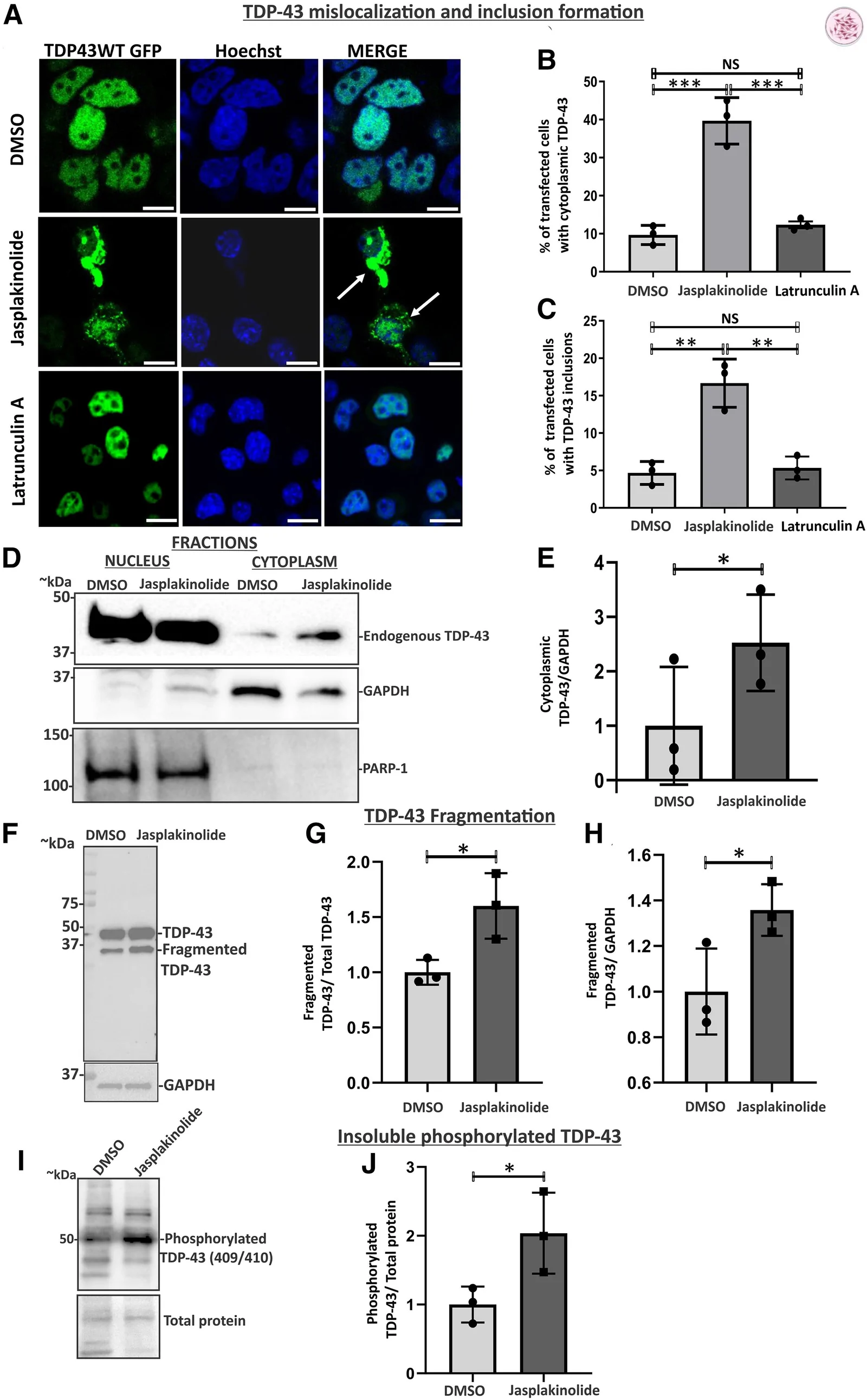
**Actin polymerization induces TDP-43 pathology in neuronal cells.** (**A**) Fluorescent confocal microscopy of GFP-tagged TDP-43^WT^ expressing NSC-34 cells, with Hoechst staining. White arrows represent cytoplasmic TDP-43 inclusion-like structures. Scale bar = 10 μm. (**B**) Quantification of transfected cells in **A** displaying cytoplasmically localized GFP-tagged TDP-43 after treatment with DMSO (9.67%), jasplakinolide (39.67%), or latrunculin A (12.33%), >100 cells per group were examined. Data are represented as mean ± SD, *n* = 3, one-way ANOVA, Tukey’s multiple comparison test ***P < 0.001. (**C**) Quantification of transfected cells in **A** displaying cytoplasmic GFP-tagged TDP-43 inclusions after treatment with DMSO (4.67%), jasplakinolide (16.67%) or latrunculin A (5.33%). Data are represented as mean ± SD, *n* = 3, one-way ANOVA, Tukey’s multiple comparison test **P < 0.005. (**D**) Western blotting of nuclear and cytoplasmic fractions prepared from untransfected NSC-34 cells treated with DMSO and jasplakinolide using anti-GAPDH (as a cytoplasmic marker), anti-PARP-1 (as a nuclear marker) and anti-TDP-43 antibodies. (**E**) Quantification of cytoplasmic TDP-43 normalized to GAPDH, relative to the mean of control (DMSO) cells from the western blots in **D**, mean ± SD (*n* = 3), unpaired *t*-test, two-tailed *P < 0.05. A statistically significant increase in the levels of cytoplasmic TDP-43 in cells treated with jasplakinolide compared to DMSO was detected. (**F**) SH-SY5Y cells were treated with 1 µM jasplakinolide or DMSO for 1 h, and soluble fractions were subjected to western blotting for TDP-43 and GAPDH. More fragmented TDP-43 (35 kDa) is present in jasplakinolide-treated cells compared to DMSO control. (**G** and **H**) Quantification of fragmented TDP-43 (35 kDa) normalized to total TDP-43 [full-length TDP-43 (43 kDa) + fragmented TDP-43(43 kDa)] or GAPDH levels, relative of the mean of control cells in **F** confirms a significant increase in cells treated with jasplakinolide in both cases. Data are represented as mean ± SD, *n* = 3, unpaired *t*-test, *P < 0.05. (**I**) Insoluble fractions were prepared from SH-SY5Y cells treated with 1 µM jasplakinolide or DMSO for 1 h and analysed by western blotting for phosphorylated TDP-43. (**J**) Quantification of insoluble phosphorylated TDP-43 normalized to total protein, relative of the mean of control (DMSO) from western blots in **I** reveals significantly elevated levels of phosphorylated TDP-43 in the insoluble fractions of jasplakinolide-treated cells compared to DMSO. Data are represented as mean ± SD, *n* = 3, unpaired *t*-test; *P < 0.05. NS = non-significant; SD = standard deviation; WT = wild-type.Cell culture plate icon was created in BioRender. Jayakumar, M. (2026) https://BioRender.com/om4t94t.

As overexpression of TDP-43 produces high, non-physiological levels of protein, we next examined endogenous TDP-43 as a more physiological approach. Nuclear and cytoplasmic subcellular fractions were prepared from untransfected NSC-34 cells treated with 1 µM jasplakinolide or DMSO for 1 h. Western blotting for PARP-1 (nuclear marker) and GAPDH (cytoplasmic marker) (Fig. 6D) confirmed that the fractions were enriched with nucleus and cytoplasm, respectively (Fig. 6D). Quantification of TDP-43 revealed that significantly more TDP-43 was present in the cytoplasm of cells treated with 1 µM jasplakinolide compared to DMSO (Fig. 6D and E).

To confirm that actin polymerization induces TDP-43 pathology, additional features were examined using endogenous TDP-43: misfolding, fragmentation and hyperphosphorylation.^4,42^ A different cell line was used to confirm that findings were not cell line- or species-dependent (mouse versus human). Human neuronal SH-SY5Y cells were treated with 1 µM jasplakinolide or DMSO for 1 h. Western blotting revealed that both full-length and fragmented TDP-43 were present in soluble fractions in each case, consistent with previous observations that DMSO can induce TDP-43 fragmentation.^43,44^ However, the proportion of fragmented TDP-43 (35 kDa) normalized to total TDP-43 was significantly increased in jasplakinolide-treated cells compared to DMSO (Fig. 6F and G). Similarly, fragmented TDP-43 normalized to GAPDH was significantly increased in jasplakinolide-treated cells compared to DMSO (Fig. 6F–H).

TDP-43 phosphorylation is enriched in insoluble cellular fractions, reflecting its pathological aggregation state.^4^ Therefore, TDP-43 phosphorylation and aggregation were examined by preparing insoluble cell fractions. Western blotting using an antibody specific for Ser 409/410 TDP-43 phosphorylation revealed significantly more phosphorylated, insoluble TDP-43 in jasplakinolide-treated cells compared to DMSO (Fig. 6I and J). Furthermore, jasplakinolide did not induce generalized nucleocytoplasmic transport defects. Following immunocytochemistry for importin-β1, neither DMSO nor jasplakinolide produced any obvious defects. Importin-β1 displayed similar levels of localization and fluorescence intensity at the nuclear envelope. Hence our findings are not explained by disruption of nucleocytoplasmic transport (Supplementary Fig. 8E–G). Hence these results demonstrate that actin polymerization induces TDP-43 mislocalization, cytoplasmic inclusions, hyperphosphorylation and aggregation, representing hallmark features of TDP-43 pathology.

### Actin polymerization recruits TDP-43 into SGs

We next examined whether inducing actin polymerization pharmacologically promotes SG formation and recruitment of TDP-43 using TIAR-1, a canonical SG marker that rapidly translocate from the nucleus to the cytoplasm upon cellular stress, aggregating into SGs.^45^

NSC-34 cells were treated with either 1 µM jasplakinolide, DMSO or 1 µM latrunculin A for 1 h: GFP-alone, TDP-43^WT^, ALS-linked TDP-43^M337V^ or cytoplasmic TDP-43^ΔNLS^. Following DMSO treatment all groups displayed sparse TIAR-1–labelled SGs, reflecting low basal stress. Similarly, latrunculin A did not significantly increase SG formation. Hence simply disturbing actin dynamics is insufficient to promote SG assembly. In contrast, cells treated with jasplakinolide displayed significantly more TIAR-positive SGs in TDP-43^WT^, TDP-43^M337V^ and TDP-43^ΔNLS^ groups. Confocal imaging revealed frequent co-localization of TDP-43 within SG structures. This recruitment was evident for TDP-43^WT^, TDP-43^M337V^ and particularly TDP-43^ΔNLS^ (yellow arrows, Fig. 7A). Quantitative analysis confirmed that jasplakinolide markedly increased TIAR-1–positive SGs in TDP-43 cell populations, far exceeding DMSO and latrunculin A controls (Fig. 7B and Supplementary Fig. 9A–C). Basal SG levels were low in DMSO and latrunculin A–treated cells. In contrast, jasplakinolide induced robust SG recruitment in all TDP-43-cells: TDP-43^ΔNLS^ (30.33%), TDP-43^M337V^ (19.67%) and TDP-43^WT^ (12.67%) compared to GFP-only (6.6%). Hence stabilization of F-actin, rather than general disruption of actin filaments, drives SG assembly and TDP-43 recruitment.

**Figure 7:**
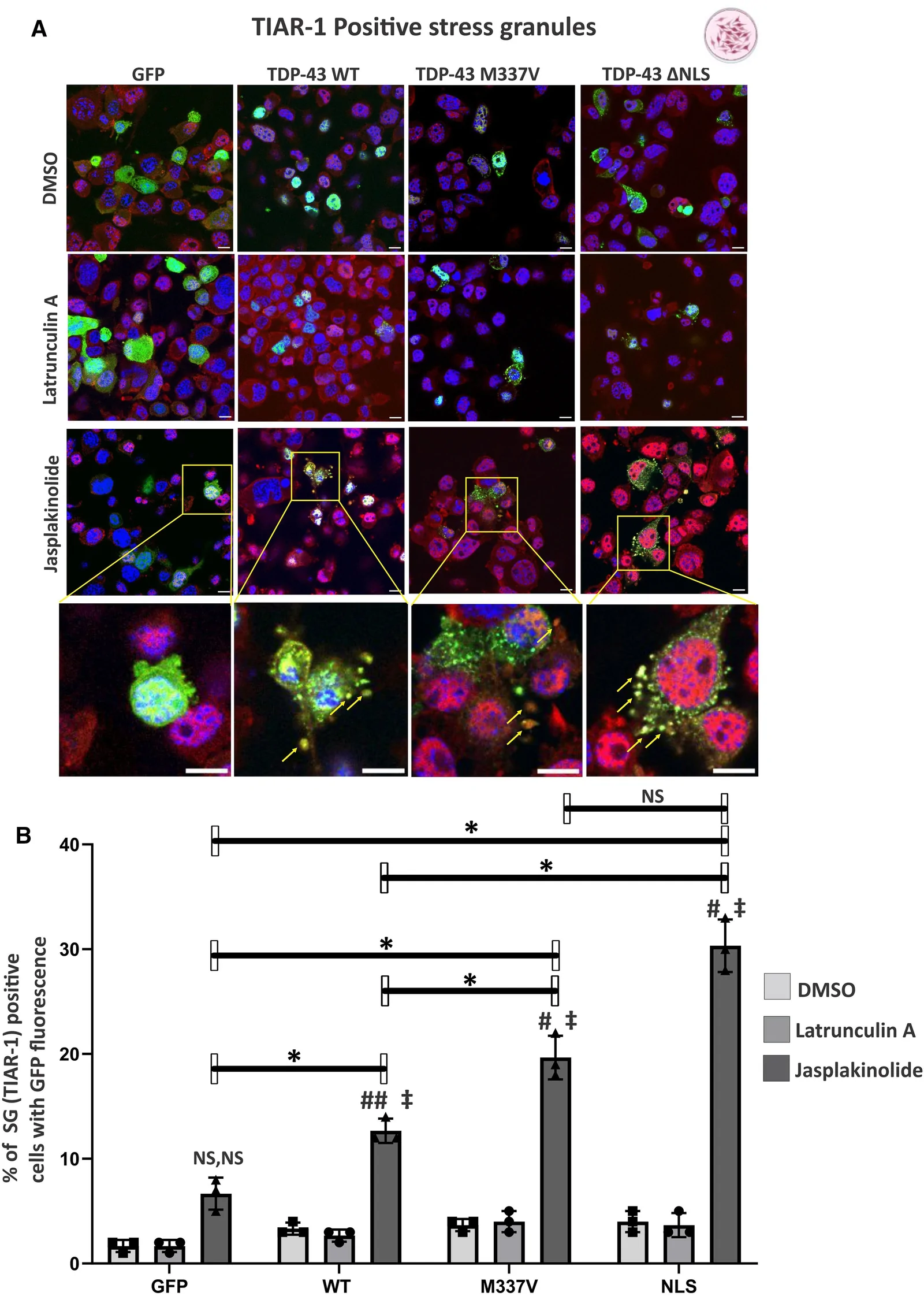
**Actin polymerization recruits TDP-43 into stress granules.** (**A**) Confocal fluorescence microscopy of NSC-34 cells expressing GFP, TDP-43^WT^-GFP, TDP-43^M337V^-GFP or TDP-43^ΔNLS^-GFP following treatment with DMSO, 1 µM latrunculin A or 1 µM jasplakinolide for 1 h. Cells were immunostained with TIAR-1 (red) and counterstained with Hoechst (blue). SGs containing TDP-43 (co-localized green and red signal) are indicated by yellow arrows. Insets show high-magnification views of SGs illustrating clear co-localization of TDP-43 with TIAR-1. Scale bars = 10 μM. (**B**) Quantification of the percentage of GFP-positive cells containing TIAR-1–positive SGs in the cells in **A**. Jasplakinolide treatment significantly increased SG formation in all TDP-43 expressing cells compared to DMSO cells (GFP: 1.67%; TDP-43^WT^: 3.3%; TDP-43^M337V^: 3.66%; TDP-43^ΔNLS^: 4%) and latrunculin A control cells (GFP: 1.67%; TDP-43^WT^: 2.67%; TDP-43^M337V^: 4%; TDP-43^ΔNLS^: 3.67%). In contrast, jasplakinolide induced robust SG recruitment in all TDP-43–expressing cells, with the highest proportion observed in the cytoplasmic TDP-43^ΔNLS^ variant (30.33%), followed by TDP-43^M337V^ (19.67%) and TDP-43^WT^ (12.67%), whereas GFP-alone controls remained at basal levels (6.6%). Data are presented as mean ± SD (*n* = 3 independent experiments; >100 cells analysed per condition); two-way ANOVA with Tukey’s multiple comparison test: *P < 0.05.. ^#^Statistical significance compared to DMSO: ^#^*P* < 0.05, ^##^*P* < 0.005. ^‡^Statistical significance compared to latrunculin A: ^‡^*P* < 0.05. NS = non-significant; SD = standard deviation; SG = stress granule; WT = wild-type.Cell culture plate icon was created in BioRender. Jayakumar, M. (2026) https://BioRender.com/om4t94t.

To confirm these findings, we also used HuR as a second SG marker. NSC-34 cells treated with jasplakinolide (1 µM, 1 h) displayed more HuR-positive SGs compared with DMSO whereas latrunculin A induced far fewer SGs that did not differ from DMSO-treated cells (Supplementary Fig. 9D and E). These results confirm that actin polymerization drives SG formation. We next examined whether these SGs recruit TDP-43. Confocal imaging revealed HuR and TDP-43 co-localized with SGs, with jasplakinolide significantly increasing the proportion of SG-positive cells containing TDP-43 compared with DMSO and latrunculin A (Supplementary Fig. 9F and G). Thus, actin polymerization promotes both SG formation and TDP-43 recruitment into SGs.

### Cofilin S3A peptide reduces pathological features of TDP-43

To examine a more direct link between cofilin hyperphosphorylation and TDP-43 pathology, and to explore potential therapeutic strategies, we investigated the effect of cofilin-based peptides on TDP-43 pathology (Supplementary Table 4). Peptides do not readily cross membranes, so we included a sequences known to enhance permeability; a cell-penetrating peptide (RKKRRQRRR) derived from HIV transactivator of transcription (TAT)^46^ (Supplementary Table 4).

Three peptides were synthesized: (i) a control containing TAT sequence only (TAT-CTRL); (ii) a control (S3) containing the first 16 cofilin residues (MA**S**GVAVSDGVIKVFN), including the Ser3 phosphorylation site, which can be both activated/inactivated, competing with endogenous cofilin for LIMK1 phosphorylation, exerting a dominant negative effect^47^; and (iii) a constitutively active cofilin sequence (TAT+S3A) in which substitution of serine 3 to alanine (‘S3A’: MA**A**GVAVSDGVIKVFN) results in non-phosphorylatable cofilin. Notably, S3A blocks LIMK1-mediated Ser3 phosphorylation of cofilin, preventing its inactivation.^48,49^ Hence, this unphosphorylated form of cofilin remains active, promoting actin depolymerization and turnover, resulting in more G-actin. We hypothesized that because aberrant cofilin hyperphosphorylation induces TDP-43 pathology, TAT+S3A will mitigate production of pathological TDP-43.

We first confirmed that the peptides are active and modulate actin polymerization in Neuro2A cells. Cells were treated with each peptide for 24 h, and G-actin/F-actin *in vitro* assays and western blotting revealed a statistically significant increase in G/F-actin ratio in cells treated with TAT+S3A peptide compared to TAT+S3 and TAT-CTRL (Fig. 8A and B). These results confirm that S3A peptide effectively reduces actin polymerization by shifting actin dynamics towards monomeric G-actin.

**Figure 8:**
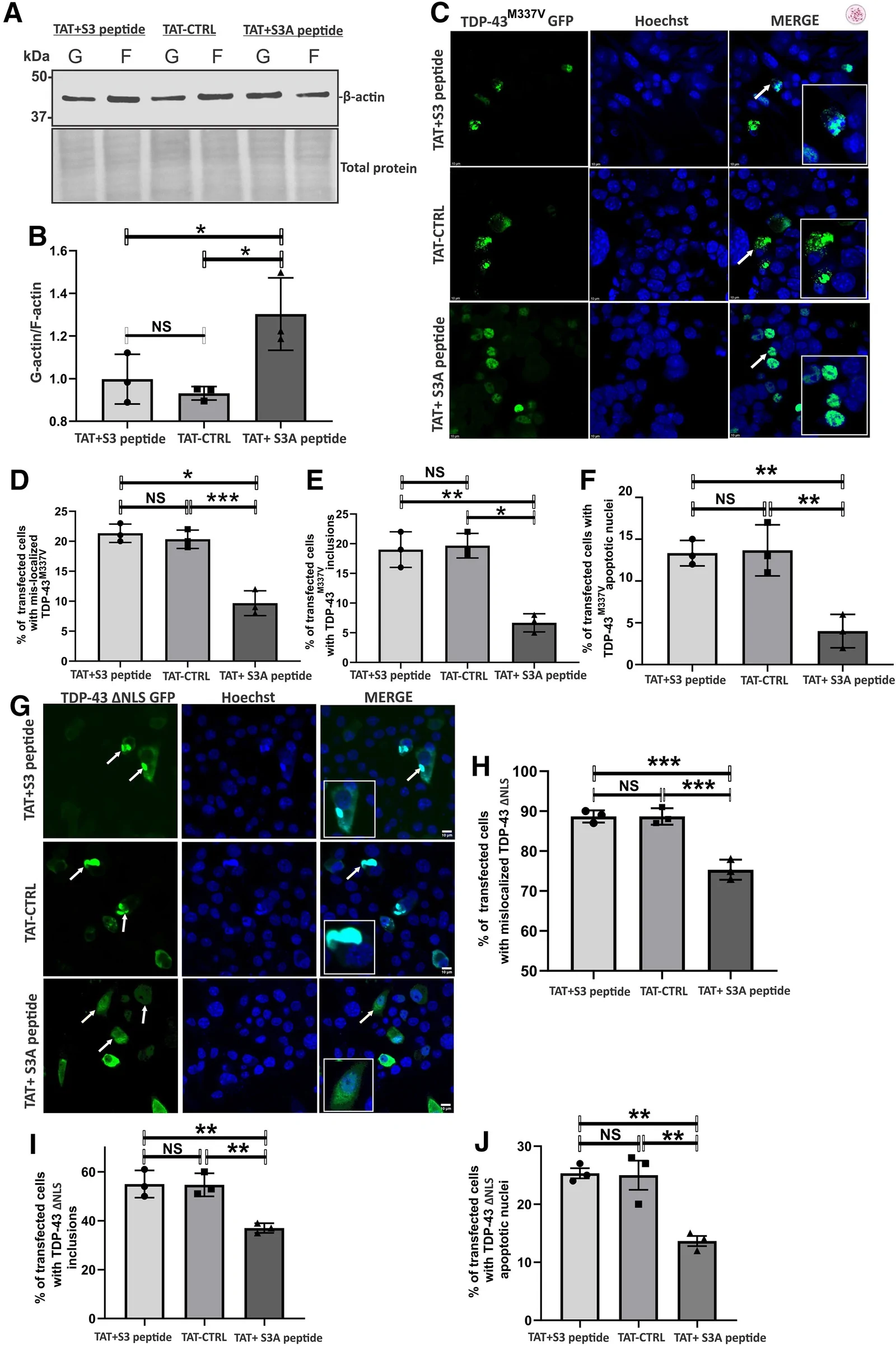
**Active cofilin S3A peptide reduce pathological mutant TDP-43 aggregation.** Neuro2A cells were treated with TAT+S3 peptide, TAT-CTRL peptide or TAT+S3A peptide for 24 h, and G-actin/F-actin fractionation assays were then performed. (**A**) Western blotting of the G-actin (G) and F-actin (F) fractions in cells treated with TAT+S3A, TAT+S3 or TAT-CTRL peptides, Ponceau S used as a loading control. (**B**) Quantification of the G/F-actin ratio normalized to total protein relative to the mean of controls from the blots in **A** shows that treatment with the TAT+S3A peptide significantly reduces actin polymerization compared to both TAT+S3 or TAT-CTRL treatment. Data are presented as mean ± SD (*n* = 3 independent experiments), one-way ANOVA Tukey’s multiple comparison test, *P < 0.05. (**C**) Confocal fluorescent microscopy images of GFP-tagged TDP-4^M337V^ expressing NSC-34 cells with Hoechst staining. White arrows represent TDP-43 inclusion-like structures present in the cytoplasm. Scale bar = 10 μm. *Inset*: Magnified image of single transfected cell. (**D**–**F**) Quantification of transfected TDP-43^M337V^ expressing cells with (**D**) cytoplasmic mislocalized TDP-43, (**E**) inclusions and (**F**) apoptotic nuclei. More than 100 cells per group were examined. Data are represented as mean ± SD (*n* = 3 independent experiments; >100 cells analysed per condition), one-way ANOVA Tukey’s multiple comparison test; *P < 0.05, **P < 0.005, ***P < 0.001. (**G**) Confocal fluorescent microscopy of GFP-tagged TDP-43^ΔNLS^-expressing Neuro2A cells treated with TAT+S3, TAT-CTRL or TAT+S3A peptides. White arrows indicate cytoplasmic TDP-43 inclusions characteristic of TDP-43^ΔNLS^ mislocalization. Scale bar = 10 μm. (**H**) Quantification of the percentage of TDP-43^ΔNLS^ expressing cells containing mislocalized cytoplasmic TDP-43 after peptide treatment. TAT+S3A significantly reduced TDP-43 mislocalization compared to both TAT+S3 and TAT-CTRL peptides. Data are presented as mean ± SD (*n* = 3 independent experiments; >100 cells analysed per condition), one-way ANOVA with Tukey’s multiple comparison test; ***P < 0.001. (**I**) Quantification of the percentage of TDP-43^ΔNLS^-expressing cells containing cytoplasmic TDP-43 inclusions after peptide treatment. TAT+S3A significantly reduced inclusion formation compared to both TAT+S3 and TAT-CTRL peptides. Data are presented as mean ± SD (*n* = 3 independent experiments; >100 cells analysed per condition), one-way ANOVA with Tukey’s multiple comparison test; **P < 0.005. (**J**) Quantification of apoptotic nuclei in TDP-43^ΔNLS^-expressing cells shows that TAT+S3A treatment significantly reduces apoptosis relative to TAT+S3 and TAT-CTRL. Data are mean ± SD (*n* = 3 independent experiments; >100 cells analysed per condition), one-way ANOVA with Tukey’s multiple comparison test; **P < 0.005. NS = non-significant; SD = standard deviation; WT = wild-type.Cell culture plate icon was created in BioRender. Jayakumar, M. (2026) https://BioRender.com/om4t94t.

Next, we examined whether the peptides were protective against TDP-43 pathology in cells expressing GFP-tagged ALS-associated TDP-43^M337V^ or TDP-43^ΔNLS^ for 24 h. Cells were treated with peptides for an additional 48 h and examined for TDP-43 mislocalization, inclusions and apoptosis (condensed nuclei, a sensitive method used previously).^50-53^ There were no significant differences in the proportion of TAT+S3 or TAT-CTRL treated cells in TDP-43^M337V^ inclusions, mislocalization or apoptotic nuclei. However, significantly fewer cells treated with TAT+S3A formed mislocalized TDP-43^M337V^, inclusions and apoptotic nuclei, compared to TAT+S3 and TAT-CTRL (Fig. 8C–F). Similarly, TAT-CTRL and TAT+S3-treated cells exhibited more cytoplasmic TDP-43^ΔNLS^ inclusions (arrows) than cells treated with TAT+S3A, which displayed more diffuse TDP-43^ΔNLS^ in the nucleus and cytoplasm (Fig. 8G). Significantly fewer TAT+S3A-treated TDP-43^ΔNLS^ cells displayed cytoplasmic mislocalization (Fig. 8H and I) and apoptotic nuclei compared to both TAT+S3 and TAT-CTRL (Fig. 8J) cells. There were no significant differences between cells treated with TAT+S3 or TAT-CTRL. Hence the constitutively active cofilin S3A peptide, lacking Ser3 phosphorylation, inhibits key features of TDP-43 pathology, confirming that aberrant cofilin phosphorylation induces TDP-43 pathology. These data also suggest therapeutic approaches based on cofilin phosphorylation may be effective in ALS.

## Discussion

Here we identify hyperphosphorylation of cofilin as a novel pathogenic mechanism associated with SALS, the most common form of ALS. It is present in MNs from SALS spinal cords and TDP-43 rNLS8 mice, thus in the most relevant cell type in ALS. This leads to the production of more F-actin. Hyperphosphorylation of LIMK1 and elevation of Tpm-4.1/4.2 in SALS and rNLS8 spinal cords enhance cofilin phosphorylation and produce more F-actin, providing mechanistic insights into these events.

We also show that pharmacological stabilization of F-actin induces TDP-43 pathology (mislocalization, inclusion formation, hyperphosphorylation, fragmentation) and promotes recruitment into SGs. The cofilin peptide studies also link TDP-43 pathology to cofilin phosphorylation. Hence aberrant cofilin hyperphosphorylation disrupts actin dynamics, driving TDP-43 pathology and SG recruitment in SALS. Preventing cofilin phosphorylation may thus mitigate TDP-43 pathology in ALS. This is the first study to correlate TDP-43 pathology with altered actin dynamics in SALS.

Increased phosphorylation of cofilin was detected early, at symptom onset, and at symptomatic disease stages, in rNLS8 mice, when TDP-43 pathology is present. However, there were no differences when mislocalization of TDP-43 is incomplete and truncation and phosphorylation are not present in this model.^34^ Similarly, no differences in cofilin phosphorylation were evident at the recovery phase, when TDP-43 pathology is absent. Hence phosphorylation of cofilin correlates with human TDP-43 pathology and cytoplasmic expression of TDP-43.

More cofilin phosphorylation was previously detected in a small cohort of *C9ORF72* ALS/FTD patient induced pluripotent stem cell (iPSC)-derived neurons^38^ and at end-stage SOD1^G93A^ mice.^54^ Importantly we have shown that the increased cofilin phosphorylation we detected in SALS is not due to *C9ORF72* haploinsufficiency. Dysregulated actin dynamics is also linked to rare familial ALS cases via profilin mutations.^33^ Hence, together these findings imply that aberrant cofilin phosphorylation may be common to different forms of ALS.

Disrupting actin dynamics in SALS would have important consequences because it is connected to many cellular processes relevant to ALS including axonal transport,^55^ protein quality control,^56^ integrated stress response (ISR),^57^ maintenance of redox conditions,^26^ nucleocytoplasmic shuttling,^58^ DNA damage^28,59^ and apoptosis.^60^ Actin also supports many fundamental processes in neurons; morphogenesis and structural plasticity, axonal/dendritic initiation, axonal growth, guidance, branching and dendritic spine formation.^61^ Hence, our findings link cofilin/actin dysregulation to multiple cellular processes relevant to ALS.

Jasplakinolide induced the formation of SGs containing TDP-43. SGs are thought to be initially protective, but during chronic stress they persist and seed the aggregation of TDP-43.^62^ Cofilin and actin polymerization have a major role in translation during the ISR,^57,63^ a central signalling network that sensors specific types of stress via four kinases.^57,63^ The phosphorylation of LIMK1/2 and cofilin is triggered by activation of Rho-family small GTPases (RhoA, Rac1 and Cdc42). Activation of RhoA stimulates ROCK, whereas Rac1 and Cdc42 activate PAK.^64^ Both kinases phosphorylate and activate LIMK1/2, leading to phosphorylation of cofilin and inhibition of its actin-severing function.^11,65^ The ISR is regulated by phosphorylation of eukaryotic initiation factor 2α (eiF2α), which in turn is modulated by protein phosphatase 1 subunit complex (PPP1R15). G-actin associates with PPP1R15 and reduced G-actin induces and fine-tunes the ISR by limiting formation of the PPP1R15–eIF2α holophosphatase complex, thereby maintaining eIF2α phosphorylation and prolonging translational repression.^57,63^ This cascade results in F-actin stabilization and depletion of the G-actin pool. Consequently, activation of the RhoA–ROCK–LIMK–cofilin axis may be a crucial upstream event linking cytoskeletal dysregulation to SG formation and TDP-43 mislocalization in ALS. Persistent Rho-GTPase signalling and aberrant cofilin phosphorylation could therefore amplify cellular stress responses and contribute to neurodegeneration in SALS. The reduced G-actin levels in ALS may therefore activate the ISR and induce cellular stress in ALS.

Oxidative stress also induces SG formation and is widely implicated in ALS pathophysiology.^66^ Interestingly, cofilin undergoes oxidation and forms intercellular tunnelling nanotubes in response to oxidative stress, inducing mitochondrial dysfunction and neuronal cell death.^67,68^ This positions cofilin as a critical link between oxidative damage, neuronal pathology and intercellular communication.

TDP-43 normally shuttles between the nucleus and cytoplasm through pores in the nuclear membrane,^40^ but pathological TDP-43 disrupts nucleocytoplasmic shuttling of proteins and mRNA^69^ through cytoplasmic aggregation and mislocalization of nucleoporins and transcription factors.^69^ This perturbs the morphology of the nuclear membrane and nuclear pore complexes (NPCs).^69^ Defects in nucleocytoplasmic transport are widely described in ALS/FTD.^1^ Cofilin maintains the architecture of the nuclear membrane,^70^ and disruption to actin affects NPC integrity.^71^ Likewise, SG formation also disturbs nucleocytoplasmic transport and induces neurodegeneration in ALS.^72^ It is therefore possible that dysregulation of cofilin in ALS impairs nucleocytoplasmic transport by disrupting actin dynamics and inducing nucleocytoplasmic shuttling defects in ALS, although this was not examined here. However, increased actin polymerization induced by jasplakinolide led to mislocalization of TDP-43 to the cytoplasm, demonstrating that aberrant nucleocytoplasmic shuttling of TDP-43 is associated with dysregulation of actin dynamics in ALS.

We show the constitutively active cofilin S3A peptide is protective against ALS mutant TDP-43^M337V^ and TDP-43^ΔNLS^. These findings suggest that selectively modulating its phosphorylation state is a novel therapeutic approach for SALS that can prevent TDP-43 pathology. Further studies, particularly *in vivo* in TDP-43 mouse models, are therefore warranted in the future.

## Conclusion

In summary, this study provides novel insights into the pathophysiology of ALS/FTD, revealing that TDP-43 pathology is closely related to dysregulated cofilin phosphorylation and actin dynamics. Mechanistically we show this results from aberrant phosphorylation of LIMK1 and dysregulation of Tpm-4.1/4.2. Given its relationship to other pathways in ALS, hyperphosphorylation of cofilin links dysregulated actin dynamics, SG formation, dysregulation of nucleocytoplasmic transport and neurodegeneration in ALS/FTD. A hypothetical model illustrating the relationship between TDP-43 and dysregulation of cofilin in ALS is shown. This highlights cofilin as a central player in this complex web of biochemical alterations. Hence strategies to modulate cofilin activity, by inhibiting its phosphorylation and thus increasing its activity may be a novel therapeutic strategy in ALS, FTD and other diseases in which TDP-43 pathology is a central component.

## Supplementary Material

awag096_Supplementary_Data

## Data Availability

The data supporting the results of this study are available upon reasonable request to the corresponding author. All original western blots are provided in the Supplementary material.
