# Supplementary material for: Cofilin hyperphosphorylation triggers TDP-43 pathology in sporadic amyotrophic lateral sclerosis
Source: Brain. 2026 Mar 10;149(11):3999–4017. doi: 10.1093/brain/awag096 (PMC13730557; doi:10.1093/brain/awag096)
Supplement: Supplementary file 1 [file awag096_supplementary_data.pdf]

Aberrant cofilin hyperphosphorylation disrupts actin dynamics and triggers TDP-43 pathology in sporadic ALS

Cyril Jones Jagaraj[1], Sayanthooran Saravanabavan[1], Sonam Parakh[1], Mrithulabashini Jayakumar[1], Sara Assar Kashani[1], Sina Shadfar[1], Prachi Mehta[1], Shashi Gautam[1], Fabiha Farzana[1], Alexandra K Suchowerska[2], Kuok Yap[3], Marta Vidal[1], Audrey M.G. Ragagnin[1], Md Shafi Jamali[1], Shu Yang[1], Thomas Fath[4], David Craik[3], Julie D Atkin[1]

Author affiliations:

[1] MND Research Centre, Macquarie Medical School, Faculty of Medicine, Health and Human Sciences, Macquarie University, Sydney, NSW 2109, Australia.

[2] Translational Neuroscience Facility and Department of Physiology, School of Biomedical Sciences, University of New South Wales, Sydney, NSW, Australia

[3] Institute of Molecular Bioscience, Australian Research Council Centre of Excellence for Innovations in Peptide and Protein Science, The University of Queensland, Brisbane, QLD 4072, Australia

[4] Dementia Research Centre, Macquarie Medical School, Faculty of Medicine, Health and Human Sciences, Macquarie University, Sydney, NSW 2109, Australia.

Correspondence to
Prof. Julie D Atkin
Macquarie University
75 Talavera Road NSW 2109
Phone: 02 9850 2772

Email: julie.atkin@mq.edu.au

Running title: Cofilin hyperphosphorylation triggers TDP-43 pathology in sporadic ALS

Supplementary Figure 1

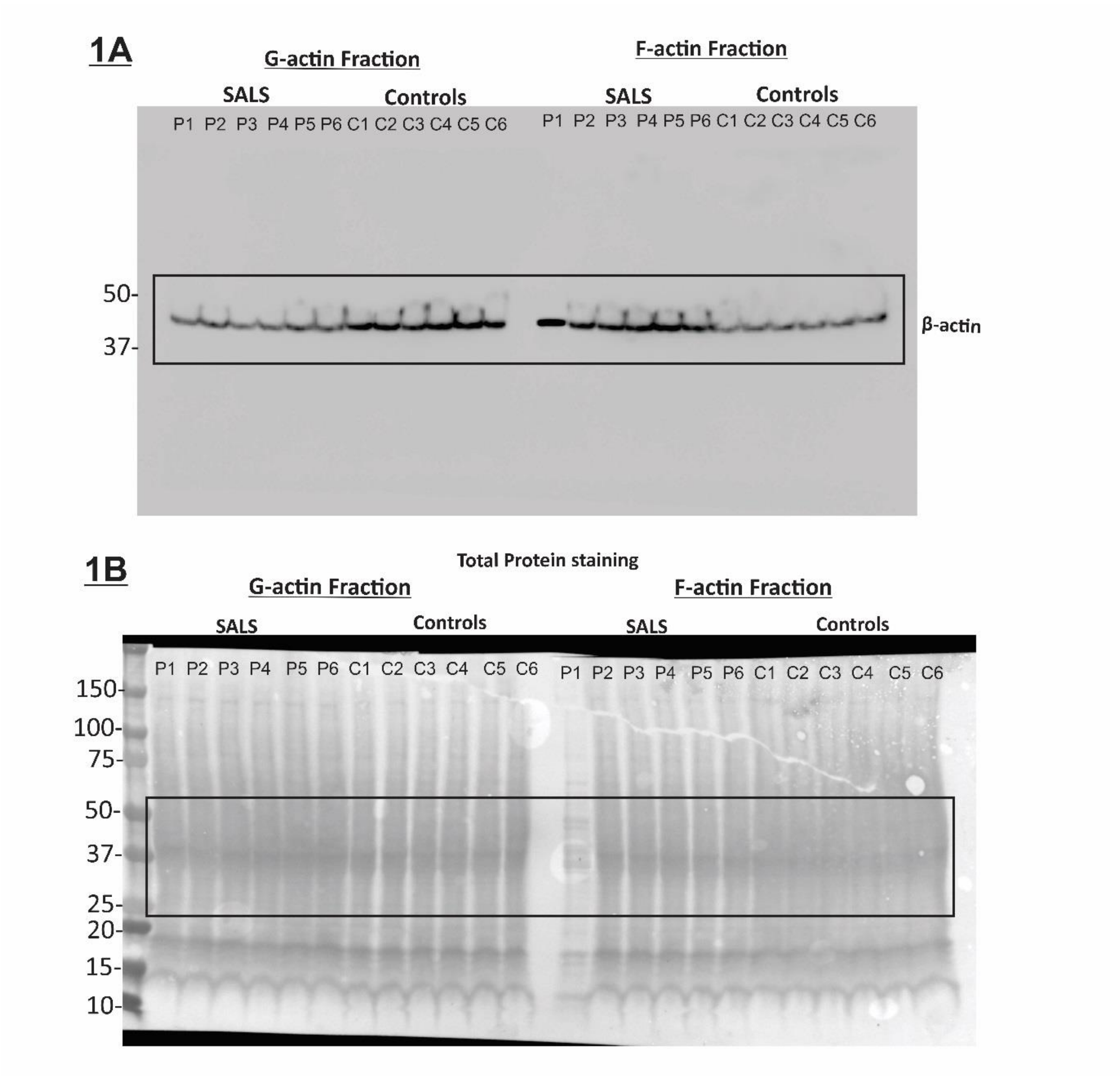

Supplementary Figure 1

(**A and B)** Full-length blots corresponding to data shown in the main **Figure 1B.** The regions highlighted by the delineated boxes are data presented in **Figure 1B.** For the ponceau stained blot (**Supp Fig 1B**), the whole lane was used to quantify the total protein present in each sample, not just the region highlighted.

Supplementary Figure 2

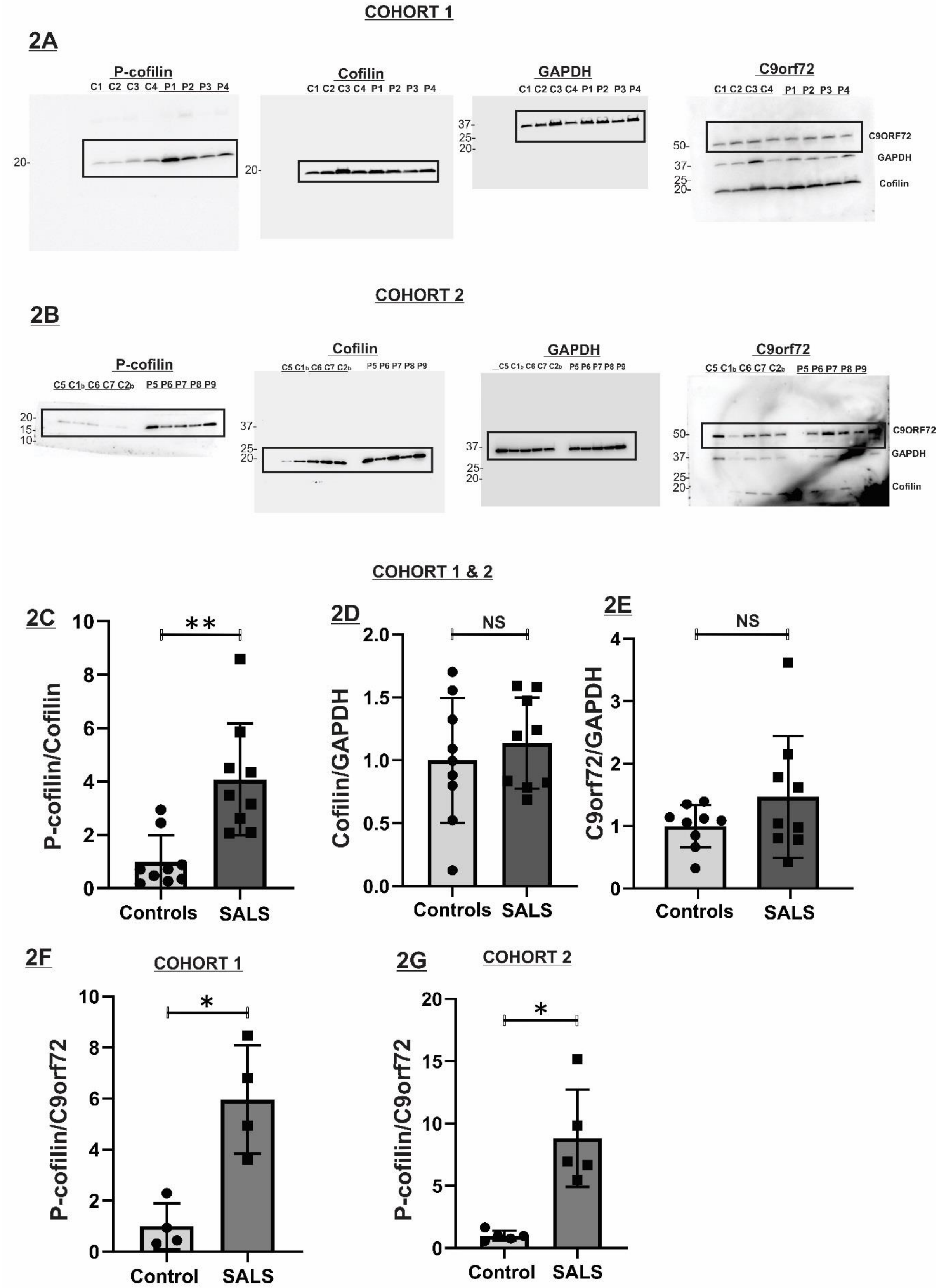

Supplementary Figure 2

(A) Full- length blots corresponding to data presented in **Figure 2A** (Cohort 1).

(B) Full- length blots corresponding to data presented in **Figure 2E** (Cohort 2).

(C) Cohort 1 and 2. Quantification of phospho-cofilin levels normalised to total cofilin, relative to the mean of control samples. Statistically significant differences in the levels of p-cofilin compared to total cofilin were observed, unpaired t-test with Welch's correction, two-tailed $**p<0.005$ (cohort 1 and 2- combined).

(D) Cohort 1 and 2. Quantification of total cofilin levels in both cohorts analysed together normalised to GAPDH, relative to the mean of control samples. No significant differences in the levels of total cofilin compared to GAPDH were observed (cohort 1 & 2- combined), unpaired t-test with Welch's correction, two-tailed, NS- non-significant. Blots were reprobed with p-cofilin, cofilin, GAPDH and C9orf72 antibodies.

(E) Cohort 1 and 2. Quantification of C9orf72 levels in both cohorts analysed together, normalised to GAPDH, relative to the mean of control samples. No significant differences in the levels of C9orf72 compared to GAPDH were observed (cohort 1 and 2- combined). unpaired t-test with Welch's correction, two-tailed, NS- non-significant. Blots were reprobed with p-cofilin, cofilin, GAPDH and C9orf72 antibodies.

(F) Cohort 1. Quantification of phosphorylated cofilin levels normalised to total C9orf72, relative to the mean of control samples. Statistically significant differences in the levels of phosphorylated cofilin compared to total C9orf72 were observed, unpaired t-test with Welch's correction, two-tailed $*p<0.05$ (cohort 1).

(G) Cohort 2. Quantification of phosphorylated cofilin levels normalised to total C9orf72, relative to the mean of control samples. Statistically significant differences in the levels of phosphorylated cofilin compared to total C9orf72 were observed, unpaired t-test with Welch's correction, two-tailed $*p<0.05$ (cohort 2).

Supplementary Figure 3

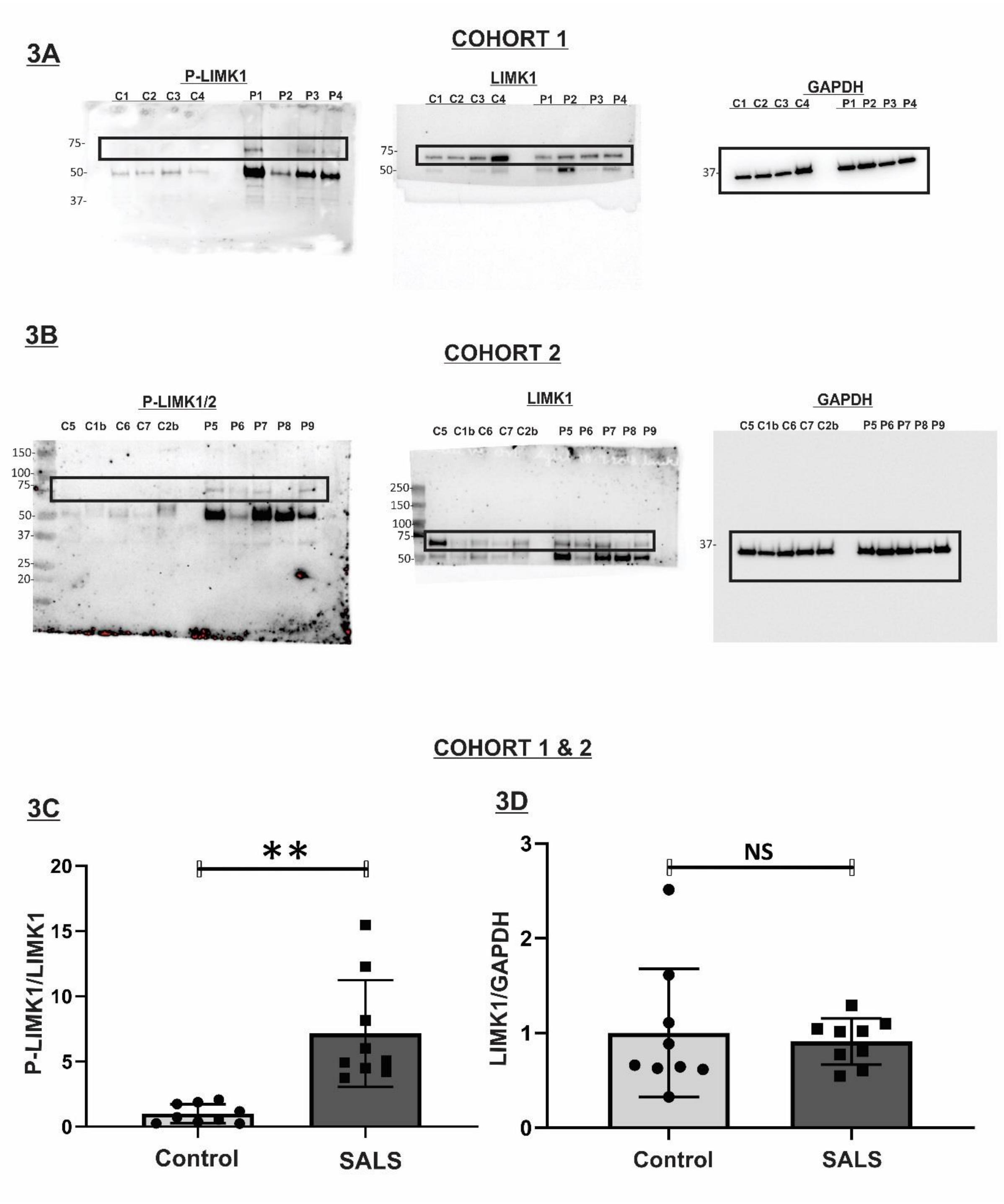

Supplementary Figure 3

(A) Full- length blots corresponding to data presented in **Figure 3A** (Cohort 1)
(B) Full- length blots corresponding to data presented in **Figure 3D** (Cohort 2)

(C) Cohort 1 & 2. Quantification of phospho-LIMK1 levels normalised to total LIMK1, relative to the mean of control samples. Statistically significant differences in the levels of p-LIMK1 compared to total LIMK1 were observed when both cohorts were analysed together -unpaired t-test with Welch's correction, two-tailed, ** $p<0.005$ (Cohort 1 & 2-combined).

(D) Cohort 1 & 2. Quantification of total LIMK1 normalised to GAPDH, relative to the mean of control samples. No significant differences in the levels of total LIMK1 compared to GAPDH were observed (Cohort 1 & 2- combined), unpaired t-test with Welch's correction, two-tailed, NS- non-significant.

Supplementary Figure 4

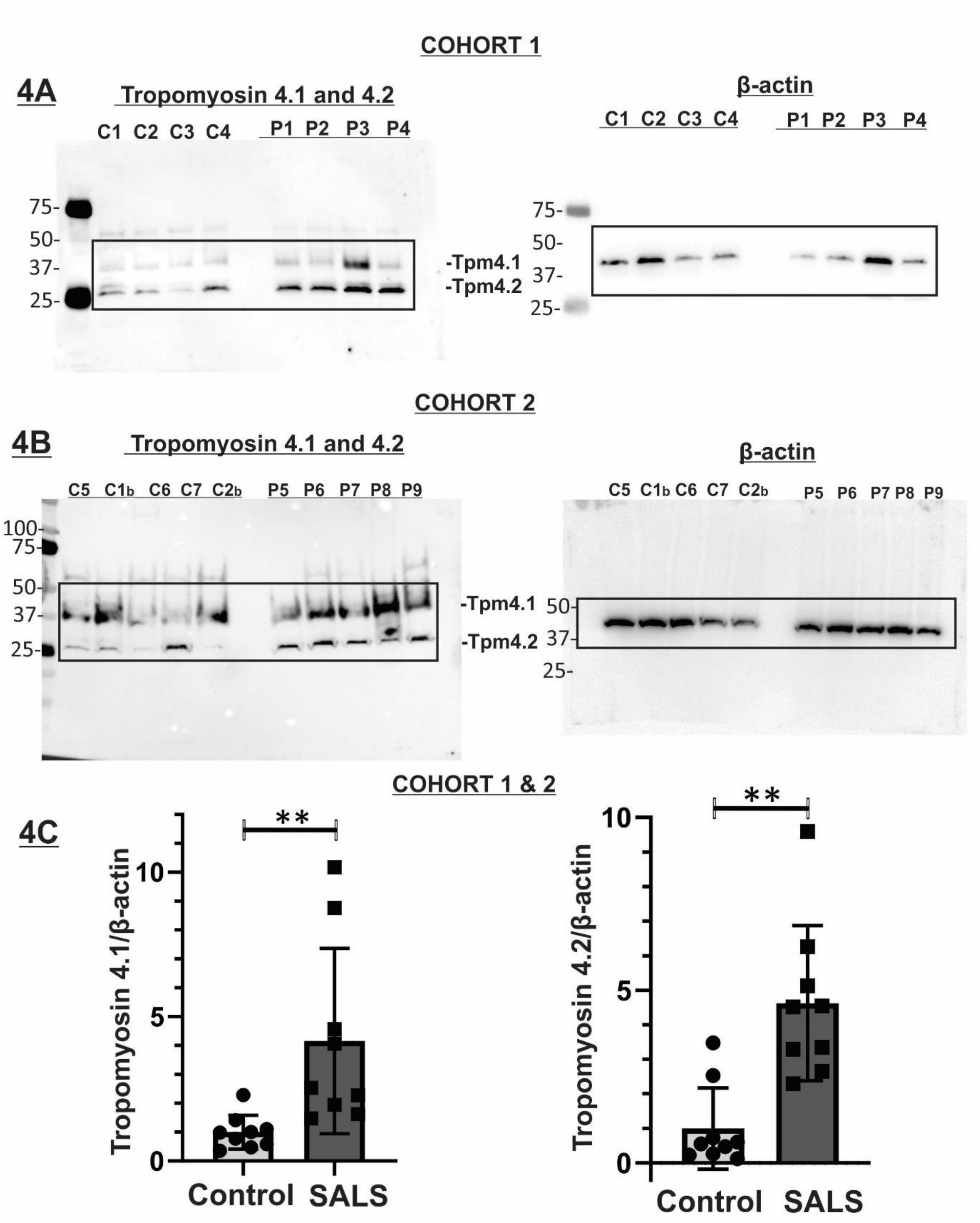

Supplementary Figure 4

(A) Full- length blots corresponding to data presented in **Figure 3G** (Cohort 1)

(B) Full- length blots corresponding to data presented in **Figure 3J** (Cohort 2). The expected molecular weight ranges for these isoforms are approximately 37 kDa for Tpm 4.1 and above 25 kDa for Tpm 4.2.

(C) Cohort 1 and 2. Quantification of Tropomyosin 4.1 and 4.2 levels normalised to β-actin, relative to the mean of control samples. Statistically significant differences in the levels of tropomyosin 4.1 and 4.2 compared to β-actin were observed, unpaired t-test with Welch's correction, two-tailed, $^{**}p<0.005$. (cohort 1 and 2 combined).

<u>Supplementary Figure 5</u>

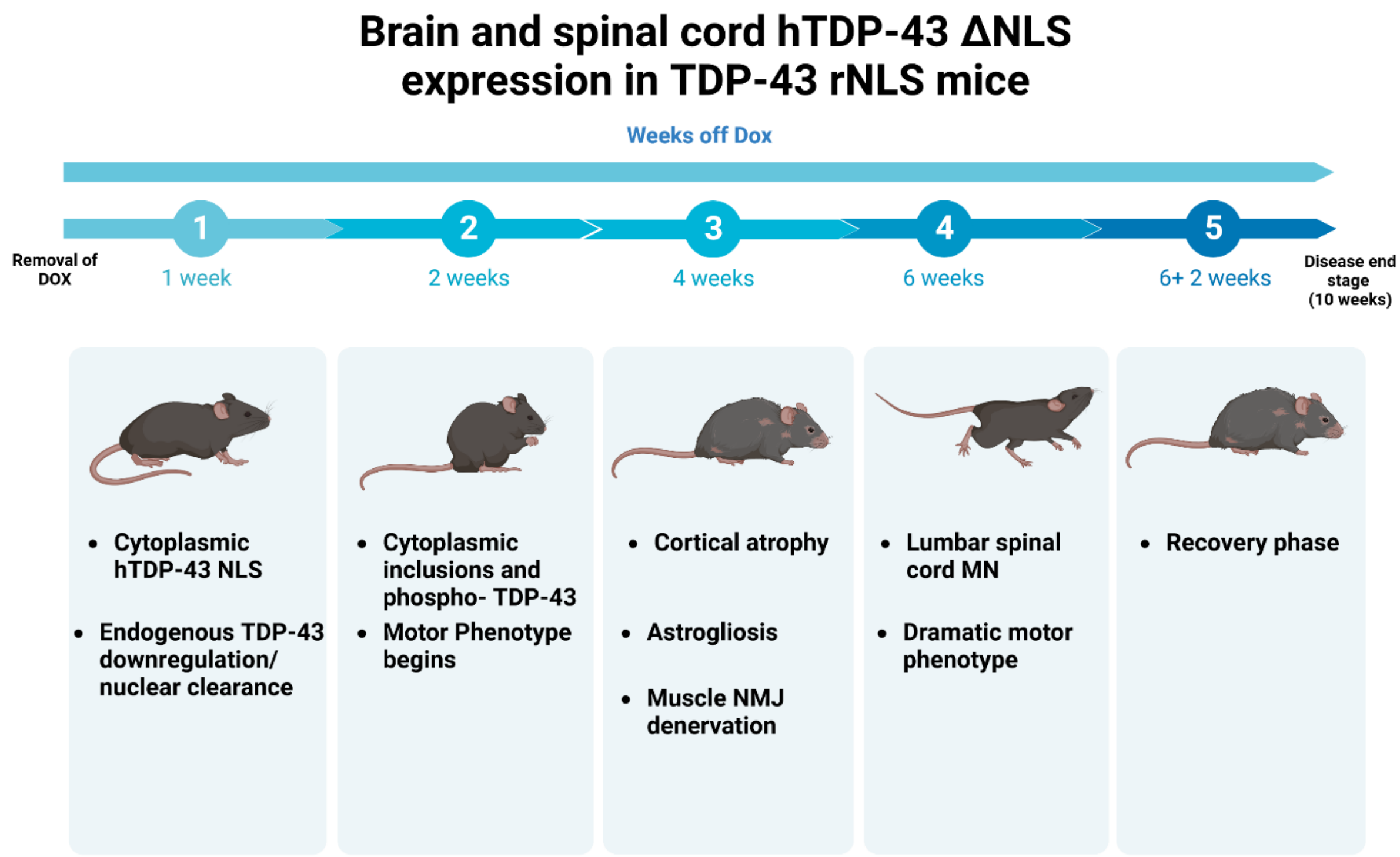

<u>Supplementary Figure 5</u>

(A) Schematic diagram illustrating disease course in TDP-43 rNLS8 mice. One week off Dox: Pre-symptomatic stage Cytoplasmic expression of TDP-43 begins. Two weeks off Dox: Symptom onset A motor phenotype begins. Four weeks off Dox: Early Symptomatic stage Mice display decreased cortical thickness accompanied by astrogliosis, indicating neurodegeneration. Six weeks off Dox: Late symptomatic stage A dramatic motor phenotype, TDP-43 pathology and loss of 30% of lumbar spinal cord motor neurons occurs. Dox re-introduced: Recovery stage: Re-introduction of Dox for two weeks eliminates cytoplasmic phosphorylated TDP-43, leading to decreased cortical atrophy. Created in BioRender. Shadfar, S. (2026) https://BioRender.com/w6alnzz.

Supplementary Figure 6

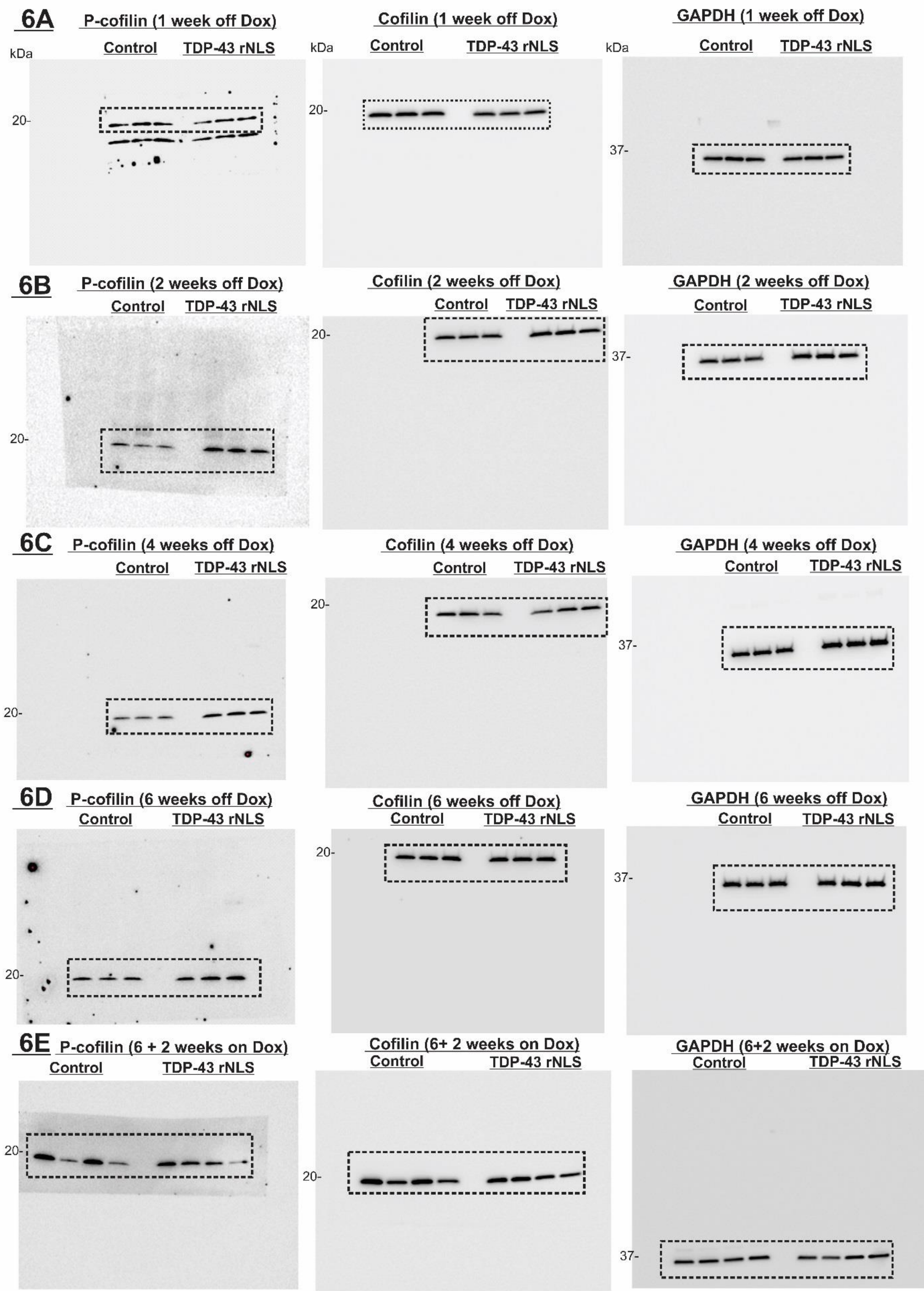

6A
P-cofilin (1 week off Dox)
kDa
Control
TDP-43 rNLS
20-
Cofilin (1 week off Dox)
kDa
Control
TDP-43 rNLS
20-
GAPDH (1 week off Dox)
kDa
Control
TDP-43 rNLS
37-
6B
P-cofilin (2 weeks off Dox)
Control
TDP-43 rNLS
20-
Cofilin (2 weeks off Dox)
Control
TDP-43 rNLS
20-
GAPDH (2 weeks off Dox)
Control
TDP-43 rNLS
37-
6C
P-cofilin (4 weeks off Dox)
Control
TDP-43 rNLS
20-
Cofilin (4 weeks off Dox)
Control
TDP-43 rNLS
20-
GAPDH (4 weeks off Dox)
Control
TDP-43 rNLS
37-
6D
P-cofilin (6 weeks off Dox)
Control
TDP-43 rNLS
20-
Cofilin (6 weeks off Dox)
Control
TDP-43 rNLS
20-
GAPDH (6 weeks off Dox)
Control
TDP-43 rNLS
37-
6E
P-cofilin (6 + 2 weeks on Dox)
Control
TDP-43 rNLS
20-
Cofilin (6+ 2 weeks on Dox)
Control
TDP-43 rNLS
20-
GAPDH (6+2 weeks on Dox)
Control
TDP-43 rNLS
37-

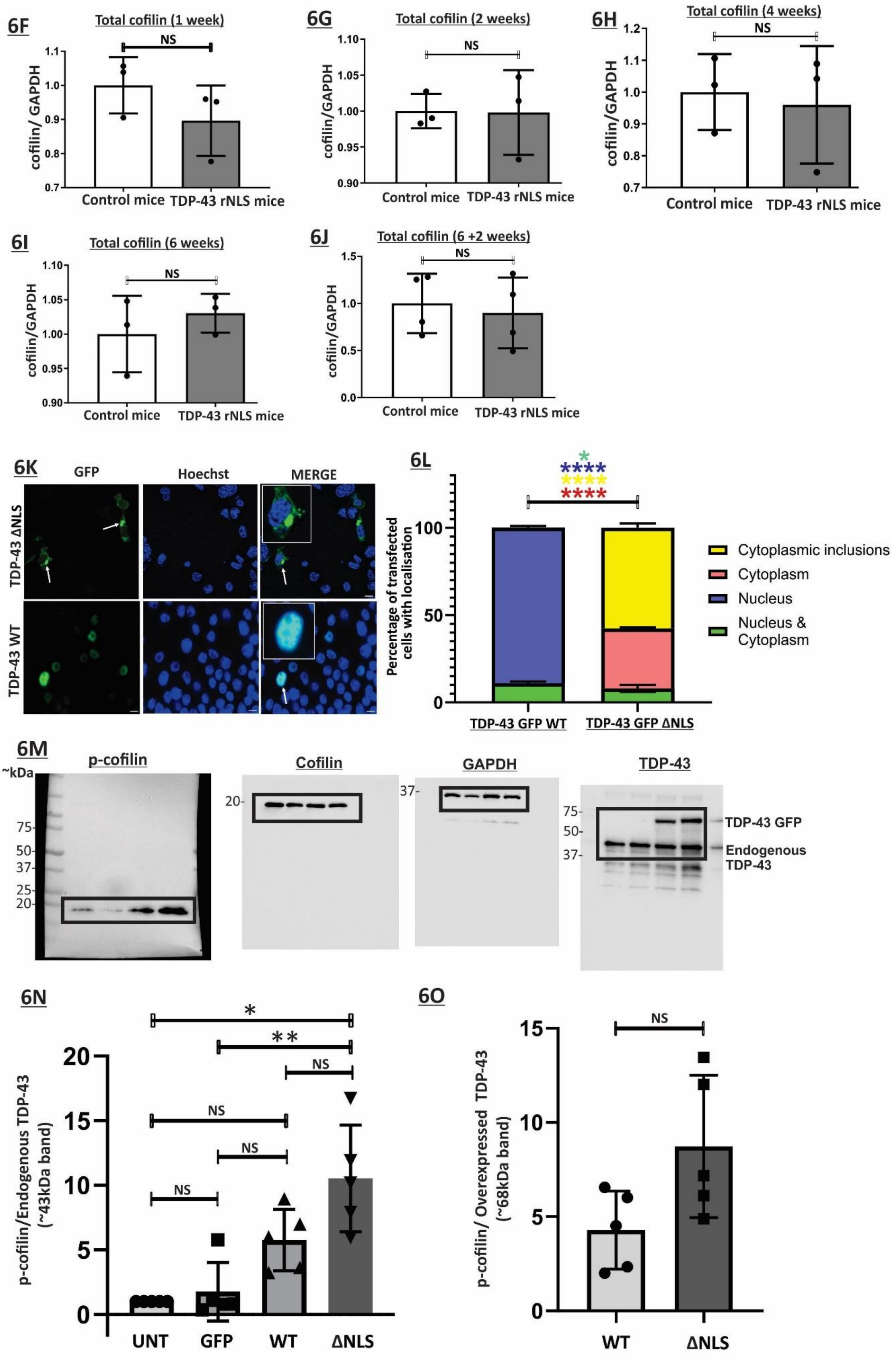
6F
Total cofilin (1 week)
NS
cofilin/ GAPDH
Control mice
TDP-43 rNLS mice
6G
Total cofilin (2 weeks)
NS
cofilin/GAPDH
Control mice
TDP-43 rNLS mice
6H
Total cofilin (4 weeks)
NS
cofilin/GAPDH
Control mice
TDP-43 rNLS mice
6I
Total cofilin (6 weeks)
NS
cofilin/GAPDH
Control mice
TDP-43 rNLS mice
6J
Total cofilin (6 +2 weeks)
NS
cofilin/GAPDH
Control mice
TDP-43 rNLS mice
6K
GFP
Hoechst
MERGE
TDP-43 ΔNLS
TDP-43 WT
6L
Percentage of transfected cells with localisation
Cytoplasmic inclusions
Cytoplasm
Nucleus
Nucleus & Cytoplasm
TDP-43 GFP WT
TDP-43 GFP ΔNLS
6M
p-cofilin
~kDa
Cofilin
GAPDH
TDP-43
TDP-43 GFP
Endogenous TDP-43
6N
p-cofilin/Endogenous TDP-43 (~43kDa band)
NS
UNT
GFP
WT
ΔNLS
6O
p-cofilin/ Overexpressed TDP-43 (~68kDa band)
NS
WT
ΔNLS

Supplementary Figure 6

(A, B) Full- length blots corresponding to data presented in **Figures 4A and 4C.**

(C, D, E) Full-length blots corresponding to data presented in **Figures 4E, 4G and 4I.**

(F, G, H, I, J) Quantification of total cofilin levels normalised to GAPDH, relative to the mean of control mice in 1 week off Dox **(Figure 4A)**, 2 weeks off Dox **(Figure 4C)**, 4 weeks off Dox **(Figure 4E)**, 6 weeks off Dox **(Figure 4G)** and recovery stage (6 weeks off Dox +2 weeks on Dox) **(Figure 4I),** mean ± SD (n = 3), *NS,* non-significant. No significant differences in the levels of total cofilin compared to GAPDH were observed, unpaired t-test, two-tailed, NS- non-significant. Blots were reprobed for p-cofilin, cofilin and GAPDH.

(K and L) Localisation of TDP-43 expressed in NSC-34 cells: Quantification of transfected NSC-34 cells expressing wild-type TDP-43 GFP or TDP-43ΔNLS GFP based on the localisation of TDP-43. Insets shows magnified image of single transfected cell. Cells were categorised as either cytoplasmic inclusions, diffuse cytoplasm only or both nuclear and cytoplasm, which included cells with TDP-43 localised in both the nucleus and cytoplasm and those with nuclear TDP-43 only. Scale bar = 10 µm. A total of >100 cells per group were examined. Data are represented as mean ±SD, n=3, two-way Anova, Sidak's multiple comparison test ***p<0.001, *p<0.05 (colored stars represent multiple comparisons between each type of TDP-43 localisation for both wild-type TDP-43 GFP and TDP-43ΔNLS).

(M) Full-length blots corresponding to data presented in **Figure 4M**

(N and O) Quantification of phosphorylated cofilin levels normalised to endogenous TDP-43(N) and overexpressed TDP-43 (O), relative to the mean of control. Data are represented as mean ±SD, n=5, one-way Anova, Kruskal-Wallis multiple comparison test (N) *p<0.05, **p<0.005, NS – non-significant and unpaired t-test (O) *NS – non-significant.*

Supplementary Figure 7

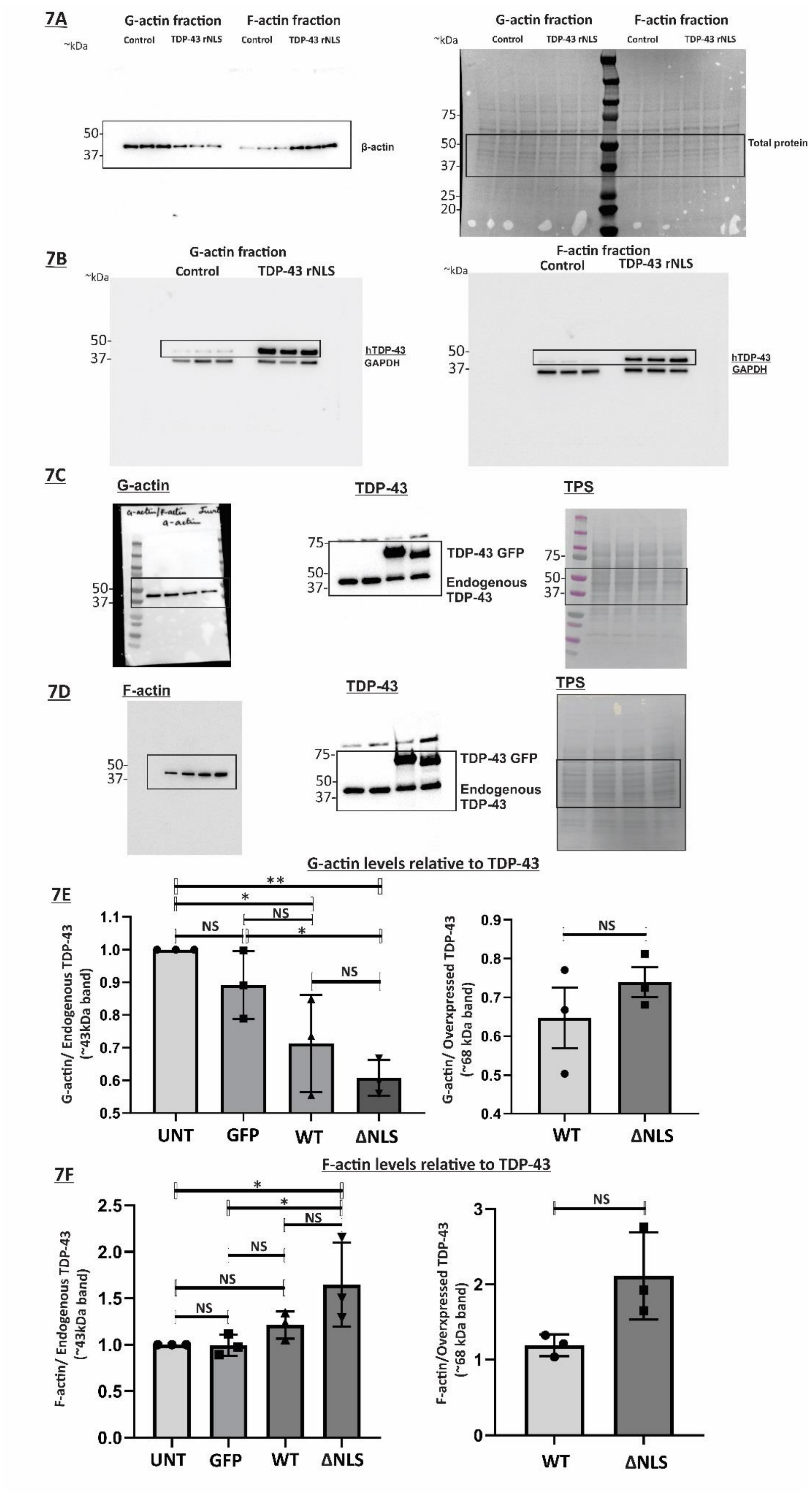

Supplementary Figure 7

(**A) Full-length blots** corresponding to data presented in **Figure 5A.** For the ponceau stained blot (**Supp Fig 7A**), the whole lane was used to quantify the total protein present in each sample, not just the region highlighted.

(B) TDP-43 blot confirms transgene expression (human TDP-43 NLS8) in TDP-43 rNLS8 mice.

(C and D) Full-length blots corresponding to data presented in **(Figure 5E and G).**For the total protein staining (TPS) (**Supp Fig 7C and D**), the whole lane was used to quantify the total protein present in each sample, not just the region highlighted.

(E) Quantification of G-actin levels normalised to endogenous TDP-43**(left graph)** and overexpressed TDP-43 **(right graph)**. Data are represented as mean ±SD, n=3, one-way Anova, Tukey's multiple comparison test *$p<0.05$, **$p<0.005$, NS – non-significant **(left graph)** and unpaired t-test, two-tailed, NS- non-significant**(right graph)**.

(F) Quantification of F-actin levels normalised to endogenous TDP-43**(left graph)** and overexpressed TDP-43 **(right graph)**. Data are represented as mean ±SD, n=3, one-way Anova, Tukey's multiple comparison test *$p<0.05$, NS – non-significant **(left graph)** and unpaired t-test, two-tailed, NS- non-significant **(right graph)**.

Supplementary Figure 8

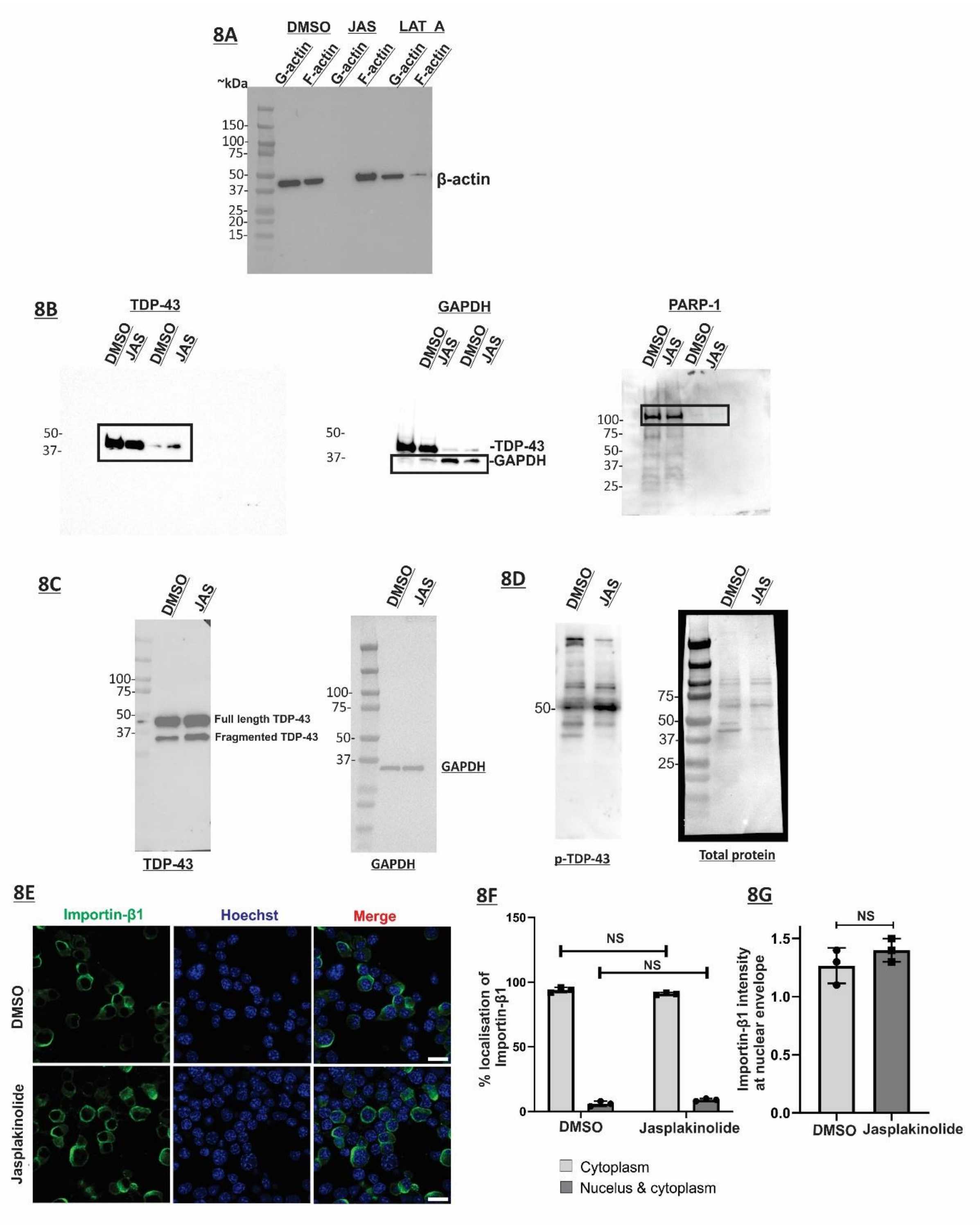

Supplementary Figure 8

(A) Jasplakinolide induces the production of F-actin and latrunculin A induces actin depolymerisation. Western blotting of G-actin and F-actin fractions from NSC-34 cells after

treatment with DMSO, 1µM jasplakinolide (JAS) or 1µM latrunculin A (LAT). Actin polymerisation is increased in NSC-34 cells treated with 1µM jasplakinolide, indicated by more F-actin compared to DMSO and latrunculin A treatment**.** In contrast, actin depolymerisation is increased in NSC-34 cells treated with 1µM latrunculin A compared to DMSO and jasplakinolide treatment.

(B) Full-length blots corresponding to data presented in **Figure 6D**

(C) Full- length blot corresponding to data presented in **Figure 6F**

(D) Full-length blots corresponding to data presented in **Figure 6I**

(E) Jasplakinolide-induced actin polymerisation does not disrupt nuclear import as assessed by importin-β localisation.

Representative confocal images of NSC-34 cells treated with DMSO (vehicle control) or jasplakinolide (1µM, 1h) and immunostained for importin-β1 (green) and counterstained with Hoechst (blue). Importin-β1 shows predominant cytoplasmic and perinuclear localisation in both DMSO- and jasplakinolide-treated cells, indicating that actin polymerisation does not cause a general defect in nuclear transport. Scale bar = 20 µm.

(F) Quantification of importin-β1 localisation following jasplakinolide treatment.

The percentage of importin-β1 signal localised in the cytoplasm and the nucleus and cytoplasm regions in NSC-34 cells treated with DMSO or jasplakinolide (1µM, 1h). Jasplakinolide treatment did not significantly alter the distribution of importin-β1, either cytoplasmic localisation (including perinuclear region) or nucleus and cytoplasmic localisation, indicating that enhanced actin polymerisation does not impair nuclear import. Data represent mean ± SD; n=3, two- way Anova, Sidak's multiple-test comparison, NS = not significant.

(G) Quantification of importin-β1 intensity at the nuclear envelope.

Mean fluorescence intensity of importin-β1 at the nuclear envelope in NSC-34 cells treated with DMSO or jasplakinolide (1 µM, 1h). Jasplakinolide treatment did not significantly change importin-β1 enrichment at the nuclear envelope, further demonstrating that actin polymerisation does not cause detectable defects in nuclear transport machinery. The fluorescent intensity per unit area at nuclear envelope was calculated and normalised to DMSO treated cells. Data represent mean ± SD; n=3, unpaired t-test, two-tailed NS = not significant.

Supplementary Figure 9

9A

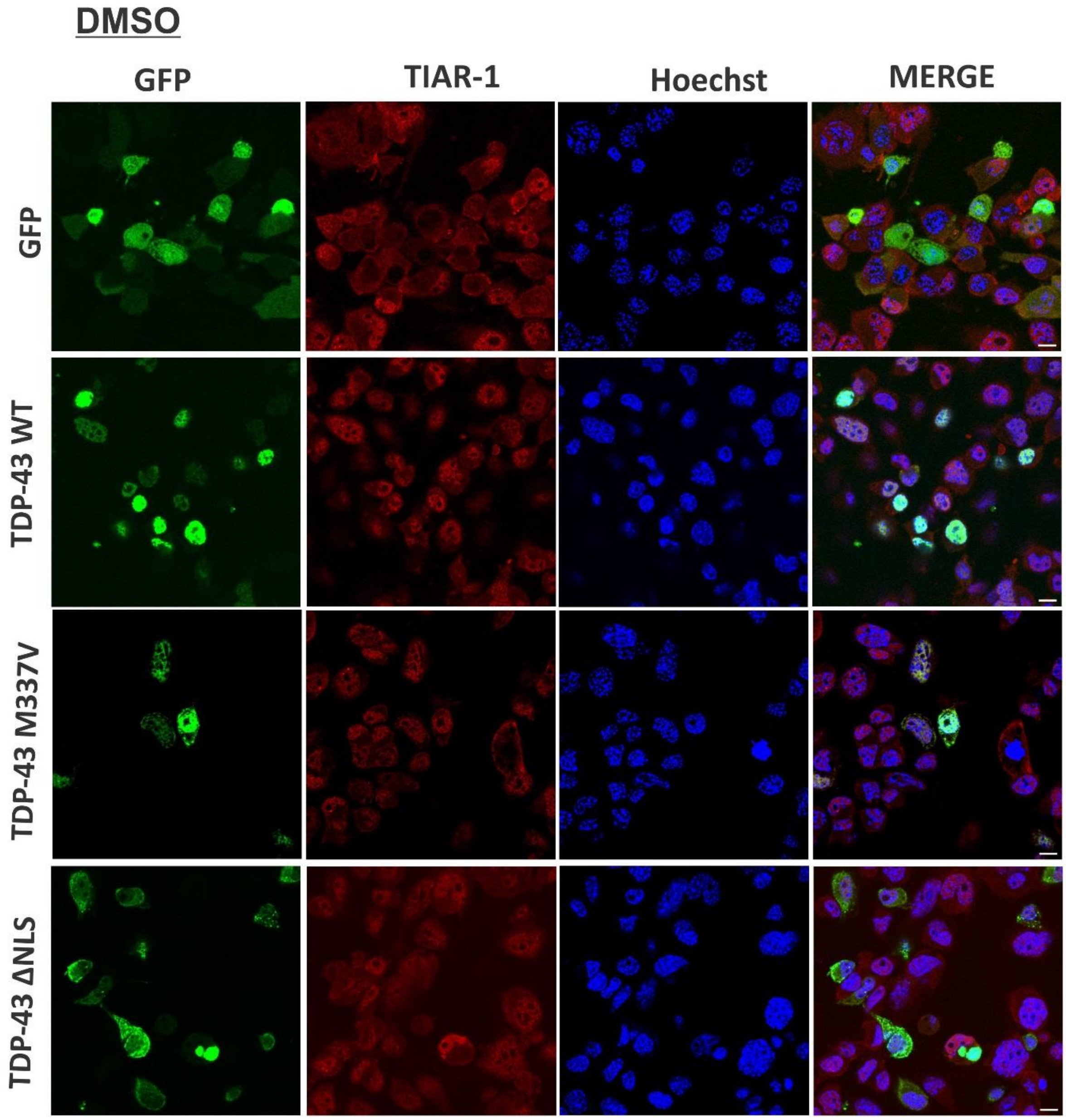

9B

LATRUNCULIN A

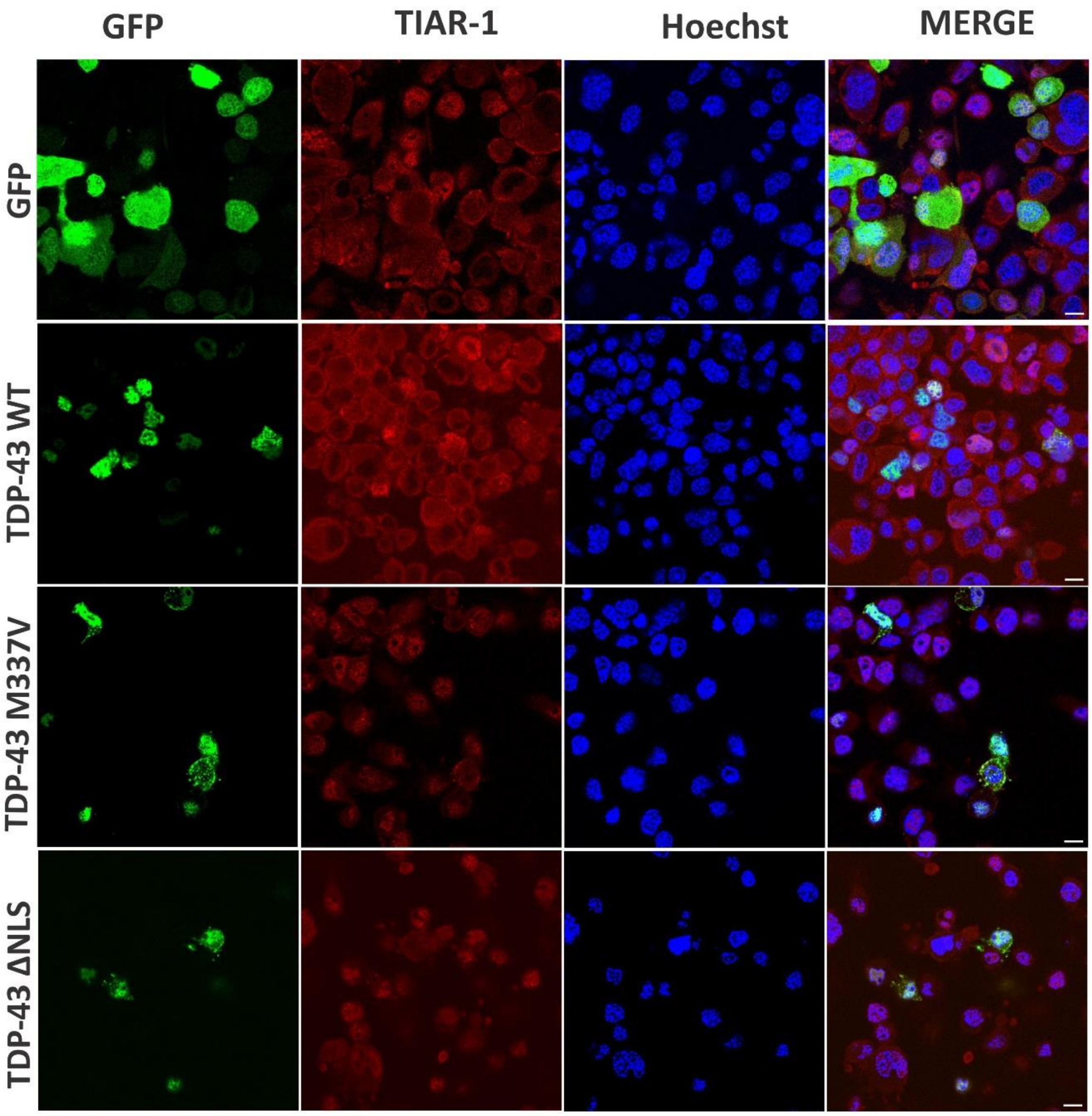

9C

JASPLAKINOLIDE

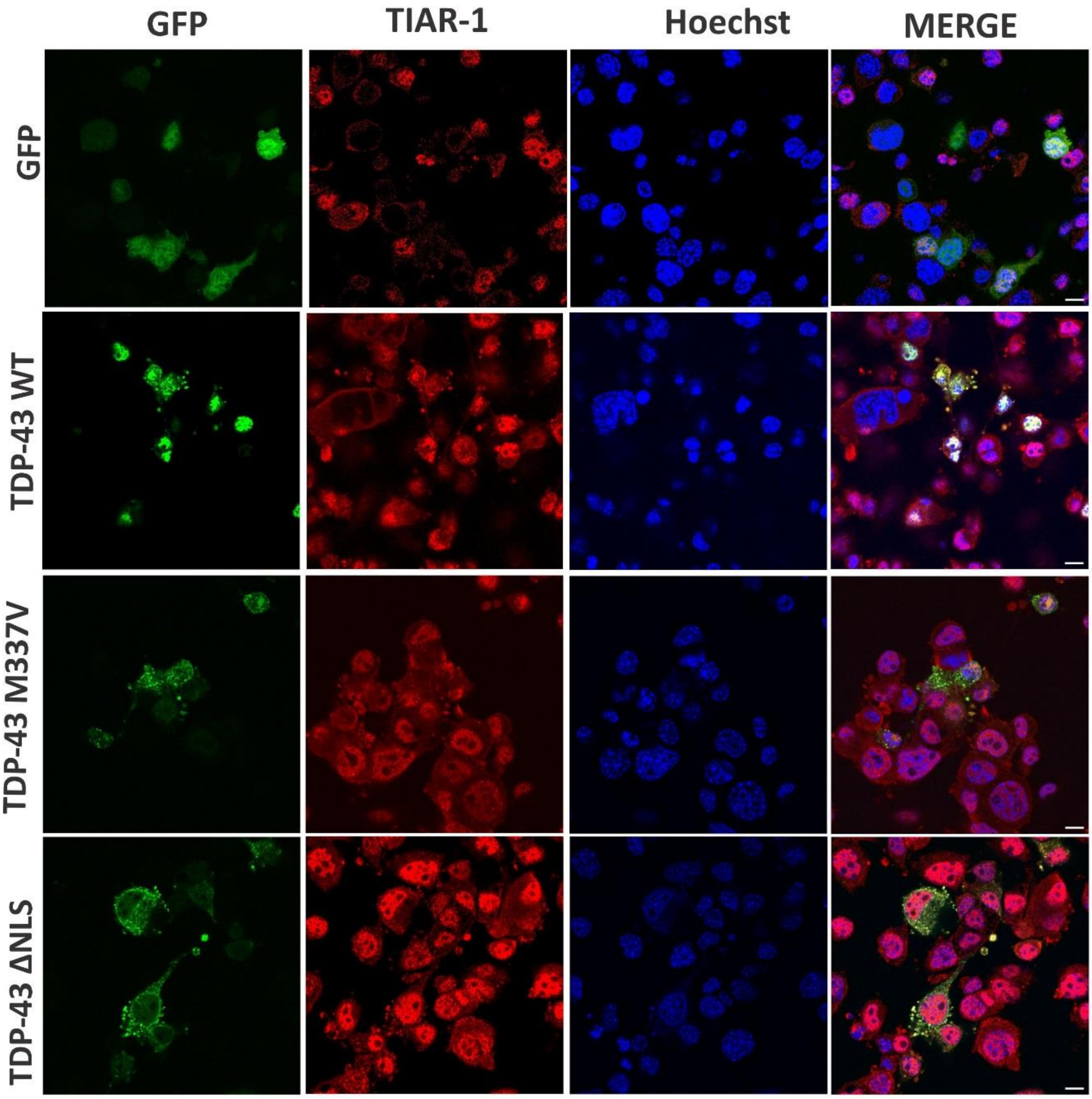

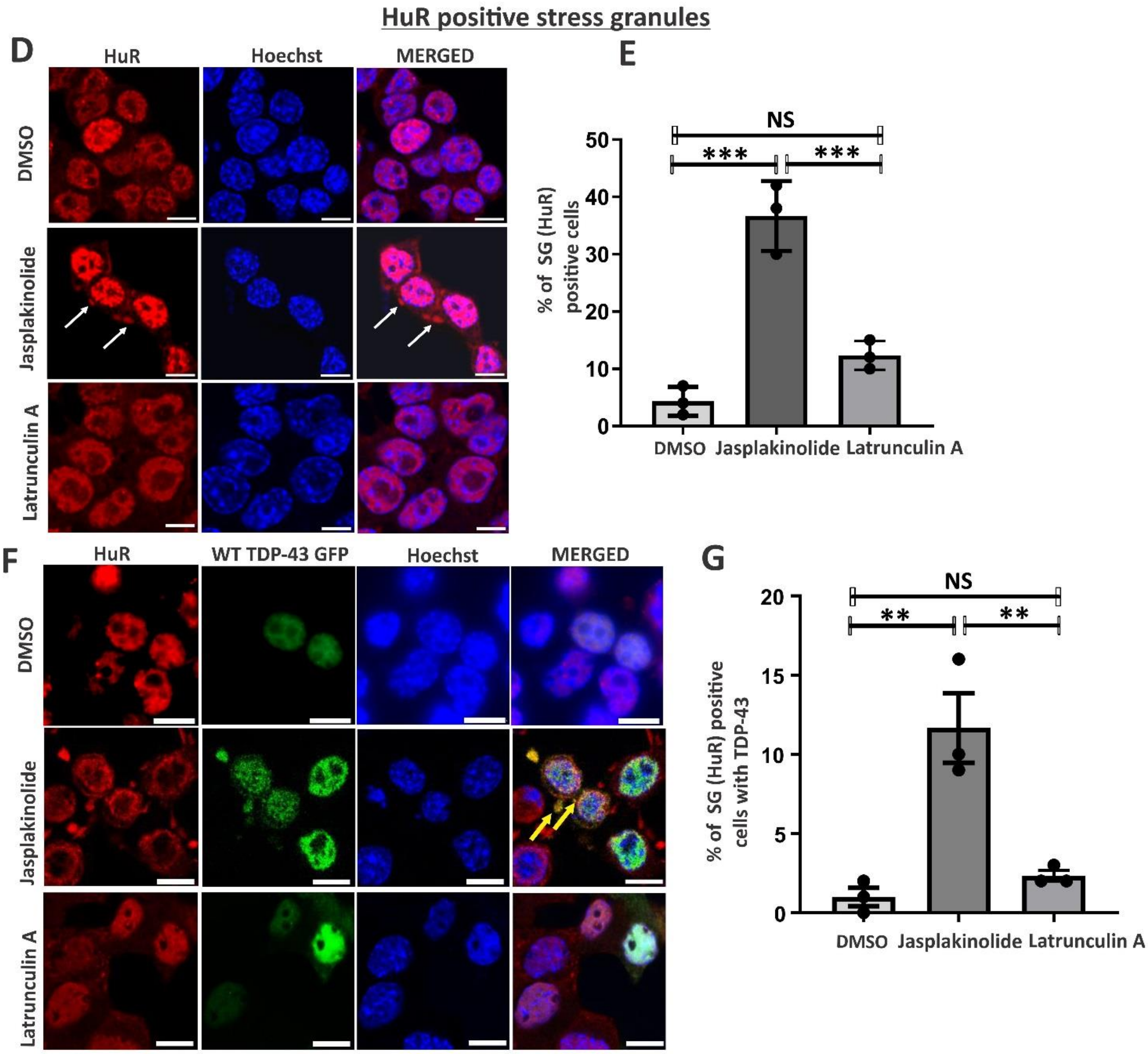

Supplementary Figure 9

(A-C) TIAR-1 staining in GFP, WT TDP-43, TDP-43^{M337V}, and TDP-43ΔNLS-expressing NSC-34 cells under different treatment conditions. Representative confocal images showing TIAR-1 (red), GFP/TDP-43 (green), and Hoechst (blue) following treatment with DMSO **(A)**, latrunculin A(**B**), or jasplakinolide **(C)**. Jasplakinolide treatment induces robust TIAR-1–positive stress granules, particularly in TDP-43–expressing cells, whereas DMSO and latrunculin A show minimal SG formation. Scale bars = 10 µm.

(D) Confocal fluorescent microscopy following HuR immunocytochemistry and Hoechst staining of NSC-34 cells (100+ cells per group) (n=3) treated with 1µM jasplakinolide, 1µM latrunculin A or DMSO. White arrows indicate HuR -positive SGs. **(E)** Quantification of cells in (D) positive for SG marker HuR after treatment with latrunculin A, jasplakinolide or DMSO. Data are represented as mean ± SD, n=3, one-way ANOVA, Tukey's multiple comparison test ***p<0.001, NS, non-significant. **(F)** Fluorescent confocal microscopy following HuR immunocytochemistry and Hoechst staining of TDP-43^{WT} GFP-tagged expressing cells treated

with 1µM jasplakinolide, 1µM latrunculin A or DMSO. Yellow arrows indicate TDP-43-positive SGs, in which TDP-43 and HuR are co-localised. White arrows indicate HuR positive stress granules. Scale bars = 10 µm. **(G)** Quantification of % of structures in (F) positive for both SG marker HuR and TDP-43 after treatment with jasplakinolide, latrunculin A or DMSO, indicating recruitment of TDP-43 to SGs. Data are represented as mean ± SD, n=3, one-way ANOVA, Tukey's multiple comparison test $^{**}p<0.005$, NS, non-significant.

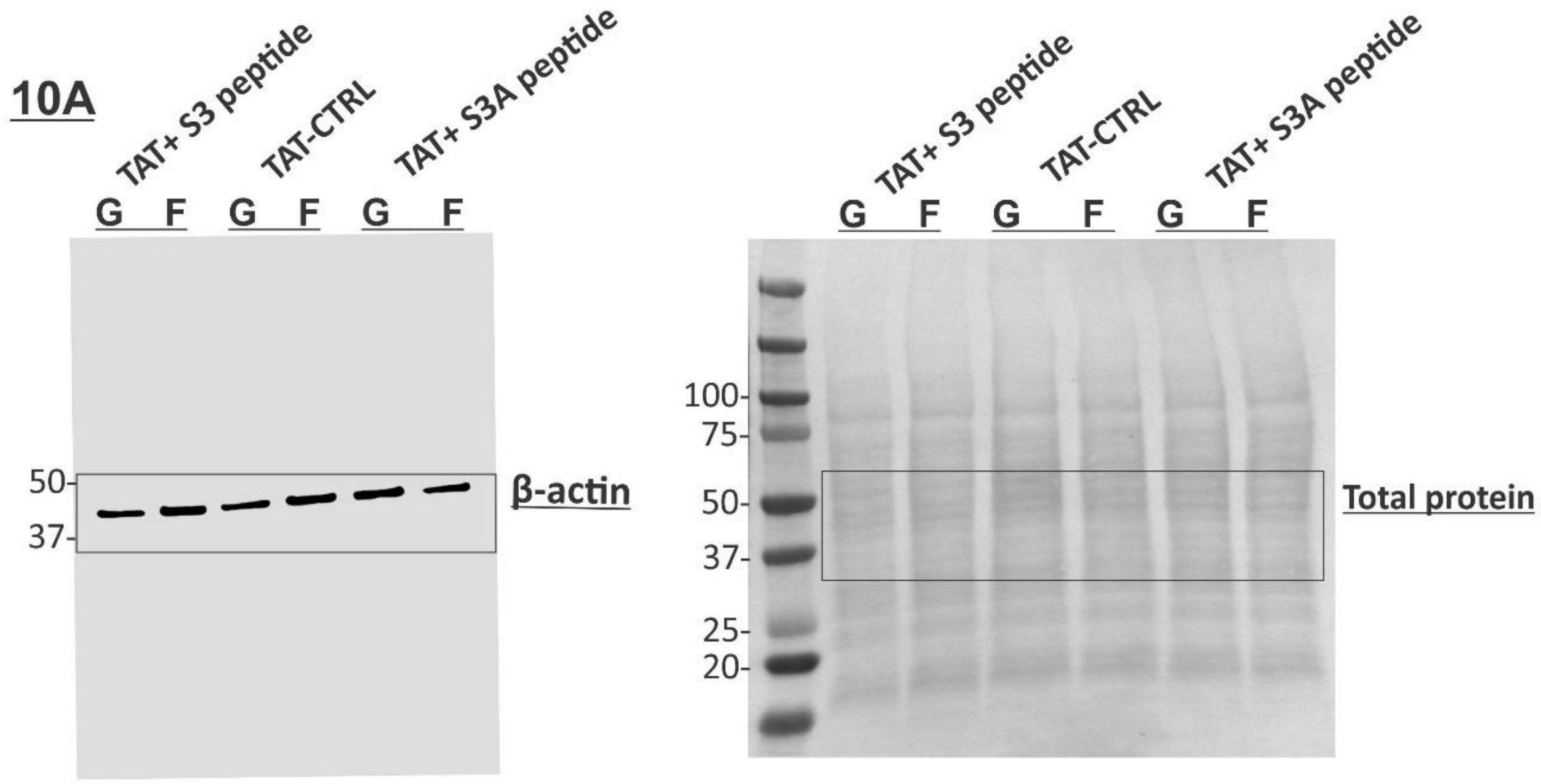

Supplementary Figure 10

(A) Full-length blots corresponding to data presented in **Figure 8A**

Supplementary Table 1: List of products, cell lines and constructs used in this study

S. No.	Product Name	Company Name	Catalogue Number
**Materials**			
1	Dulbecco's Modified Eagle Medium (DMEM	Gibco	11965092
2	Fetal Bovine Serum (FBS)	Gibco	A5670801
3	Protease and phosphatase inhibitor (PPI) cocktail	Sigma	4693159001<br>4906845001
4	Jasplakinolide	Sigma	J4580
5	Latrunculin A	Sigma	L5163
6	Hoechst 33258	Sigma	94403
7	G-Actin/F-Actin *in Vivo* Assay Biochem kit	Cytoskeleton	BK037
**Cell lines**		**Source**	
1	Neuro-2a cells mouse neuroblastoma	CellBank, Australia	
2	Undifferentiated SH-SY5Y cells human neuroblastoma	ATCC	
3	Motor neuron-like NSC-34 cells	generously provided by Professor Neil Cashman, University of British Columbia, Canada	
**Constructs**		**Source**	
1	pLenti-C-monomeric (m) green fluorescent protein (GFP) (empty vector)	Prof. Adam Walker, The University of Sydney	
2	pLenti-C-TDP-43WT-mGFP		
3	pLenti-C-TDP-43 M337V-mGFP		
4	pLenti-C-TDP-43ΔNLS-mGFP		
5	Turbo-GFP-tagged TDP-43 WT in pCMV6-AC-GFP	PMID: 26293199, PMID: 29409023	
6	Mutant TDP-43^{M337V} in pCMV6-AC-GFP	PMID: 26293199, PMID: 29409023	

<u>Supplementary Table 2 Clinical Details of Human Spinal Cord Tissues</u>

No familial history for ALS patients

Cohort 1				
**Label**	**Age**	**Gender**	**PMI (Post-mortem interval)**	**Diagnosis**
C1	52.1	Male	33	Control
C2	75.9	Male	50	Control
C3	88.6	Female	6	Control
C4	85.8	Male	71	Control
P1	63.9	Male	14	ALS
P2	84.8	Male	59	ALS
P3	82.7	Male	35	ALS
P4	75.9	Male	11	ALS
**Cohort 2**				
**Label**	**Age**	**Gender**	**PMI (Post-mortem interval)**	**Diagnosis**
***C1b**	**52.1**	**Male**	**33**	**Control**
***C2b**	**75.9**	**Male**	**50**	**Control**
C5	37.5	Male	45.5	Control
C6	63.9	Male	68	Control
**C7**	**66**	**Male**	**54**	**Control**
P5	42.8	Male	7.5	ALS
P6	53.6	Male	55	ALS
**P7**	**63.9**	**Female**	**65**	**ALS**
**P8**	**66.9**	**Male**	**21**	**ALS**
**P9**	**74.4**	**Female**	**7**	**ALS**

*C1 and C1b, and C2 and C2b, were different lysates produced from the same patient lumbar spinal cords.
Bold text – previously available RIPA lysates

Supplementary Table 3 Clinical Details of Human Spinal Cord Tissues- G/F actin assay

No familial history for ALS patients

**Label**	**Age**	**Gender**	**PMI (Post-mortem interval)**	**Diagnosis**
C1	52.1	Male	33	Control
C2	75.9	Male	50	Control
C3	88.6	Female	6	Control
C4	85.8	Male	71	Control
C5	37.5	Male	45.5	Control
C6	63.9	Male	68	Control
P1	63.9	Male	14	ALS
P2	84.8	Male	59	ALS
P3	82.7	Male	35	ALS
P4	75.9	Male	11	ALS
P5	42.8	Male	7.5	ALS
P6	53.6	Male	55	ALS

Supplementary Table 4. List of cofilin and control peptide sequence

No	Name	Peptide Sequence	Molecular weight (Monoisotopic)
1	TAT-CTRL	RKKRRQRRR	1338.87
2	TAT+ S3 peptide	RKKRRQRRRMA**S**GVAVSDGVIKVFN	2897.68
3	TAT + S3A peptide	RKKRRQRRRMA**A**GVAVSDGVIKVFN	2913.67

Supplementary Table 5: List of antibodies used in this study

S. No.	Antibodies	Catalogue No.	Company Name	Dilution
**Antibodies for Western blotting**				
1	anti-cofilin 1G6A2	66057-I-Ig	Proteintech	1:3000
2	anti-phospho-cofilin (Ser3)	#3311	CST	1:1000
3	anti-GAPDH	#60004-1-Ig	Proteintech	1:5000
4	anti-TDP-43	#TIP-TD-P09	Cosmo Biotech	1:1000
5	anti-phospho-TDP-43(409/410)	#TIP-PTD-P07	Cosmo Biotech	1:1000
6	anti-TDP-43	10782-2-AP	Proteintech	1:1000
7	anti-C9orf72	#GTX632041	GeneTex	1:1000
8	anti-phospho-LIMK1 (Thr508)	#3841	CST	1:500
9	anti-LIMK1	#3842	CST	1:1000
10	anti-β-actin	#A2228	Sigma	1:4000
11	anti- β-actin clone C4	#MAB1501	Merck	1:1000
12	anti-PARP-1	#9532	CST	1:500
13	anti-Tpm-4.2	antibody prepared in house, as previously described[40,41]	antibody prepared in house, as previously described[40,41]	1:1000
**Antibodies for Immunohistochemistry of the human spinal cord - Primary**				
1	Rabbit anti-phospho-cofilin	#3311	Cell Signaling Technology [CST]	1:100
2	Mouse anti-SMI-32	# SMI-32R	BioLegend	1:250
**Antibodies for Immunohistochemistry of the human spinal cord - Secondary**				
3	Donkey anti-rabbit Alexa Fluor 594	#A-21207	Invitrogen	1:200
4	Rabbit anti-mouse Alexa Fluor 488	#A-11059	Invitrogen	1:200
**Antibodies for Immunohistochemistry of mouse spinal cords - Primary**				
1	Rabbit anti-phospho-cofilin	#3311S	Cell Signaling Technology [CST]	1:250
2	Mouse anti TDP-43	60019-2-Ig	Proteintech	1:250
**Antibodies for Immunohistochemistry of mouse spinal cords - Secondary**				
4	Goat anti-Ms Alexa Fluor 488	A11029	Invitrogen	1:2000
5	Goat anti-Rb Alexa Fluor 647	A21244	Invitrogen	1:1000
6	Mouse anti-NeuN Alexa 555 conjugate	MAB377A5	Merck Millipore	1:100
**Antibodies for Immunocytochemistry - Primary**				
1	Mouse anti-HuR	390600	Invitrogen	1:100
2	Mouse anti-TIAR-1	610352	BD	1:100
3	Mouse anti-importin-β1	# MA3-070	Invitrogen	1:500
**Antibodies for Immunocytochemistry - Secondary**				
4	Donkey anti-mouse Alexa Flour 594	A21203	Invitrogen	1:200
5	Goat anti-Ms Alexa Fluor 488	A11029	Invitrogen	1:2000
